# Low-Dose Alteplase During Primary Percutaneous Coronary Intervention According to Ischemic Time

**DOI:** 10.1016/j.jacc.2020.01.041

**Published:** 2020-03-31

**Authors:** Peter J. McCartney, Annette M. Maznyczka, Hany Eteiba, Margaret McEntegart, Keith G. Oldroyd, John P. Greenwood, Neil Maredia, Matthias Schmitt, Gerry P. McCann, Timothy Fairbairn, Elisa McAlindon, Campbell Tait, Paul Welsh, Naveed Sattar, Vanessa Orchard, David Corcoran, Thomas J. Ford, Aleksandra Radjenovic, Ian Ford, Alex McConnachie, Colin Berry

**Affiliations:** a British Heart Foundation Glasgow Cardiovascular Research Center, University of Glasgow, Glasgow, United Kingdom; b West of Scotland Heart and Lung Center, Golden Jubilee National Hospital, Clydebank, United Kingdom; c Leeds University and Leeds Teaching Hospitals National Health Service (NHS) Trust, Leeds, United Kingdom; d South Tees Hospitals NHS Foundation Trust, Middlesbrough, United Kingdom; e Manchester University NHS Foundation Trust, Manchester, United Kingdom; f University of Leicester and the National Institute for Health Research Leicester Biomedical Research Center, Leicester, United Kingdom; g Liverpool Heart and Chest Hospital NHS Foundation Trust, Liverpool, United Kingdom; h New Cross Hospital, Royal Wolverhampton NHS Trust, Wolverhampton, United Kingdom; i Department of Hematology, Royal Infirmary, Glasgow, United Kingdom; j Department of Cardiology, Gosford Hospital, Gosford, New South Wales, Australia; k Robertson Centre for Biostatistics, Institute of Health and Wellbeing, University of Glasgow, Glasgow, United Kingdom

**Keywords:** fibrinolysis, microvascular obstruction, myocardial hemorrhage, primary percutaneous coronary intervention, ST-segment elevation myocardial infarction, AUC, area under the curve, CMR, cardiac magnetic resonance, IQR, interquartile range, MI, myocardial infarction, MVO, microvascular obstruction, OR, odds ratio, PPCI, primary percutaneous coronary intervention, STEMI, ST-segment elevation myocardial infarction, TIMI, Thrombolysis In Myocardial Infarction

## Abstract

**Background:**

Microvascular obstruction affects one-half of patients with ST-segment elevation myocardial infarction and confers an adverse prognosis.

**Objectives:**

This study aimed to determine whether the efficacy and safety of a therapeutic strategy involving low-dose intracoronary alteplase infused early after coronary reperfusion associates with ischemic time.

**Methods:**

This study was conducted in a prospective, multicenter, parallel group, 1:1:1 randomized, dose-ranging trial in patients undergoing primary percutaneous coronary intervention. Ischemic time, defined as the time from symptom onset to coronary reperfusion, was a pre-specified subgroup of interest. Between March 17, 2016, and December 21, 2017, 440 patients, presenting with ST-segment elevation myocardial infarction within 6 h of symptom onset (<2 h, n = 107; ≥2 h but <4 h, n = 235; ≥4 h to 6 h, n = 98), were enrolled at 11 U.K. hospitals. Participants were randomly assigned to treatment with placebo (n = 151), alteplase 10 mg (n = 144), or alteplase 20 mg (n = 145). The primary outcome was the amount of microvascular obstruction (MVO) (percentage of left ventricular mass) quantified by cardiac magnetic resonance imaging at 2 to 7 days (available for 396 of 440).

**Results:**

Overall, there was no association between alteplase dose and the extent of MVO (p for trend = 0.128). However, in patients with an ischemic time ≥4 to 6 h, alteplase increased the mean extent of MVO compared with placebo: 1.14% (placebo) versus 3.11% (10 mg) versus 5.20% (20 mg); p = 0.009 for the trend. The interaction between ischemic time and alteplase dose was statistically significant (p = 0.018).

**Conclusion:**

In patients presenting with ST-segment elevation myocardial infarction and an ischemic time ≥4 to 6 h, adjunctive treatment with low-dose intracoronary alteplase during primary percutaneous coronary intervention was associated with increased MVO. Intracoronary alteplase may be harmful for this subgroup. (A Trial of Low-Dose Adjunctive Alteplase During Primary PCI [T-TIME]; NCT02257294)

Primary percutaneous coronary intervention (PPCI) to emergently reopen the occluded coronary artery, restore blood flow, and secure vessel patency with a stent is the evidence-based standard of care for ST-segment elevation myocardial infarction (STEMI) (1). However, the success of PPCI is limited by failed microvascular reperfusion, which occurs in one-half of all treated patients (2,3). This complication, described as microvascular obstruction (MVO), is associated with adverse left ventricular remodeling and reduced left ventricular function and is independently predictive of cardiac prognosis (4). During PPCI, distal embolization of thrombus within the lumen of the infarct-related coronary artery and microvascular thrombosis (5, 6, 7, 8, 9), notably of fibrin-rich microthrombi (6), contribute to MVO. Myocardial hemorrhage is closely related to MVO (3) and occurs when endothelial cell injury compromises capillary integrity leading to the extravasation of blood into the extracellular space. T_2_*-weighted cardiac magnetic resonance (CMR) is the established method to identify and evaluate myocardial hemorrhage in vivo, accumulation of paramagnetic hemoglobin breakdown products leads to a shortening of T_2_* relaxation times, resulting in a hypointense zone on imaging that represents tissue hemorrhage (9,10). Late gadolinium-enhanced CMR imaging is used to identify MVO, a dark area representing failed perfusion at the core of the bright infarct. Validation in swine established that the hypointense core on T_2_* imaging corresponds with severe capillary loss and destruction resulting in tissue hemorrhage, with excellent anatomical correlation between the localization and extent of MVO and myocardial hemorrhage (9).

Fibrinolytic therapy is an effective treatment for acute coronary thrombosis (11). A facilitated PCI strategy involving full- or half-dose adjunctive fibrinolytic therapy given before PCI with stenting improves coronary flow acutely (12,13). Similarly, in patients with an expected PCI-related delay, half-dose alteplase and timely PCI improves epicardial and myocardial flow when compared with PPCI alone. However, combination-facilitated PCI involving either full-dose (14) or half-dose lytic therapy (15) causes paradoxical activation of thrombin, clot formation, and bleeding. In T-TIME (A Trial of Low-Dose Adjunctive Alteplase During Primary PCI), we hypothesized that a therapeutic strategy involving low-dose intracoronary fibrinolytic therapy with alteplase infused early after coronary reperfusion would reduce MVO. Patients with acute STEMI presenting <6 h after symptom onset and a large thrombus burden evident at initial coronary angiography were enrolled in a 3-arm dose-ranging design (10 or 20 mg of alteplase or placebo). The primary analysis determined that alteplase did not reduce the amount of MVO revealed by CMR imaging 2 to 7 days post-MI (primary outcome) and the secondary outcomes were consistent with this result (16).

Infarct size is influenced by ischemic time (17), as are the efficacies of primary reperfusion therapies, including systemic fibrinolysis (18) and primary PCI (19). In this pre-specified analysis, we hypothesized that the effects of adjunctive intracoronary administration of low-dose alteplase during PPCI could be associated with ischemic time. We assessed the associations among ischemic time, treatment group (placebo, alteplase 10 mg, alteplase 20 mg), and the primary and secondary outcomes in this clinical trial.

## Methods

### Trial design

We performed a randomized, double-blind, placebo-controlled, parallel group phase 2 clinical trial of low-dose adjunctive alteplase during PPCI, the main results of which have been published previously (16).

### Participants and eligibility criteria

Patients with a clinical diagnosis of acute STEMI with a symptom onset to reperfusion time of 6 h or less were eligible for randomization. Radial artery access was required, angiographic criteria included a proximal-mid coronary artery occlusion (TIMI [Thrombolysis In Myocardial Infarction] flow grade 0/1) or impaired coronary flow (TIMI flow grade 2) in the presence of definite angiographic evidence of thrombus (TIMI flow grade 2+) in a major coronary artery. Exclusion criteria included any contraindication to fibrinolysis or CMR and lack of informed consent. Full inclusion and exclusion criteria are described in the Supplemental Appendix.

### Setting

The participants were enrolled in 11 hospitals in the United Kingdom and guideline-based medical and invasive management was recommended (1). Enrollment started on March 17, 2016, and ended on December 21, 2017.

### Informed consent and study protocol

Screening, witnessed verbal informed consent, study drug administration, and acute assessments of efficacy took place during the standard-of-care PPCI. The protocol is included in the Supplemental Appendix. The trial had ethics committee approval, adhered to Guidelines for Good Clinical Practice in Clinical Trials (20), and complied with the Declaration of Helsinki (21).

### Randomization, implementation, and blinding

Participants were randomized by staff in the catheter laboratory using an interactive voice response–based randomization system. The randomization sequence was created using the method of randomized permuted blocks of length 6, with stratification by location of STEMI and study site. The allocation sequence was on a 1:1:1 basis among the placebo and alteplase (10 mg, 20 mg) groups and the sequence was concealed electronically. The participants, staff, and researchers were blinded to the treatment group allocation.

### Standard care

PPCI followed contemporary practice guidelines (1) (Supplemental Appendix).

### Interventions

After successful reperfusion of the infarct-related artery, the participants immediately received the allocated intervention. The study drug (placebo, alteplase 10 mg, or alteplase 20 mg) was manually infused before stent implantation. Further details are provided in the Supplemental Appendix.

### Outcomes

#### Primary outcome

The primary outcome was the amount of MVO (percentage of left ventricular mass) revealed by late gadolinium-enhanced CMR 10 to 15 min after administration of gadolinium-based contrast media. CMR at 1.5-T was scheduled during the index hospitalization, 2 to 7 days after enrollment. MVO was defined as a dark zone on early gadolinium enhancement imaging 1, 3, 5, and 7 min post-contrast injection that remained present within an area of late gadolinium enhancement at 15 min. The myocardial mass of the dark zone was quantified by manual delineation and expressed as percentage of left ventricular mass.

#### Secondary outcomes

##### Infarct definition and size

The presence of acute infarction was established based on abnormalities in cine wall motion, rest first-pass myocardial perfusion, and late gadolinium-enhancement imaging in 2 imaging planes. The myocardial mass of late gadolinium was quantified using computer-assisted planimetry and the territory of infarction was delineated using a 5-SD semi-automated method and expressed as percentage of total left ventricular mass.

##### Myocardial hemorrhage

On the T_2_* parametric maps, a threshold of 20 ms was applied. A region of reduced signal intensity within the infarcted area, with a T_2_* value of <20 ms (3,22) was considered to confirm the presence of myocardial hemorrhage. The area was manually delineated and expressed as percentage of left ventricular mass.

##### Other outcomes

Additional CMR secondary outcomes included myocardial salvage index, left ventricular end-diastolic volume, left ventricular end-systolic volume, and left ventricular ejection fraction at 2 to 7 days and 3 months, these are described in the Supplemental Appendix.

### Biochemistry

Troponin T (ng/l) area under the curve (AUC) was measured from blood samples obtained immediately before reperfusion (0 h) and then again at 2 and 24 h.

### Safety

Fibrinogen and other parameters of coagulation and hemostasis served as surrogate measures of bleeding and safety (23,24). These parameters were measured in blood samples when site logistics permitted blood sample collection. The sampling time points were at baseline before reperfusion (0 h) and 2 and 24 h post-reperfusion.

### Trial coordination

An independent Data and Safety Monitoring Committee and a Trial Steering Committee had oversight of the trial and liaised with the sponsor. Each committee had a charter that was established before enrollment started.

### Sample size and statistical methods

The sample size and statistical methods are described in detail in the Supplemental Appendix. To summarize, outcomes were analyzed using linear or logistic regression models. Continuous outcomes were transformed when necessary to improve model fit. Analyses treating randomized treatment as a 3-level or as a 2-level categorical variable (active vs. placebo) were performed. On the assumption that any treatment effects would manifest themselves as dose-dependent trends, randomized treatment was modeled as a linear trend across dose groups (0 mg, 10 mg, 20 mg) in an attempt to maximize power. The decision to model as a linear trend across treatment groups was made post hoc with knowledge of the data. All models were adjusted for the location of MI (anterior/nonanterior), as per the stratification of the randomization schedule. Models for coagulation and hemostasis parameters included an additional adjustment for baseline value (transformed in the same way as the outcome measurement). Models included ischemic time categorized in 3 groups (<2 h, ≥2 but <4 h, ≥4 to 6 h), and an interaction between ischemic time and randomized treatment.

All tests were 2-tailed, and p values <0.05 were considered significant. All statistical analyses were carried out with R version 3.2.4 (R Development Core Team 2015, Vienna, Austria) (25) according to a pre-specified statistical analysis plan. No adjustments have been made for multiple testing in these analyses, which should be viewed as exploratory rather than definitive.

## Results

**Figure 1:**
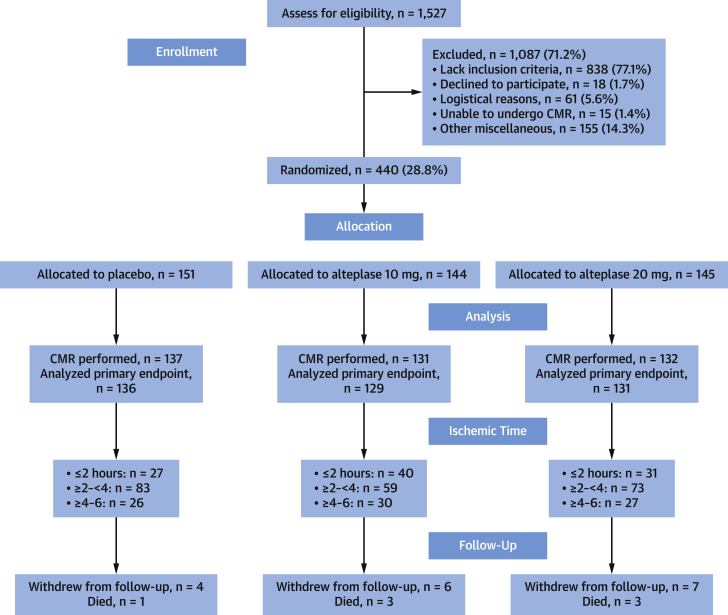
T-TIME Flow Diagram The participants are grouped by treatment group and ischemic time. Two patients (1 randomized to placebo and 1 randomized to 10 mg alteplase) received 20 mg alteplase because an incorrect treatment pack had been selected. Four patients were unable to complete the CMR examination meaning evaluable data for the primary outcome was not available: placebo group (n = 1); 10 mg–alteplase group (n = 2); 20 mg–alteplase group (n = 1). CMR = cardiac magnetic resonance; T-TIME = A Trial of Low-Dose Adjunctive Alteplase During Primary PCI.

On the recommendation of the Data and Safety Monitoring Committee, recruitment was discontinued on December 21, 2017, based on a pre-specified futility analysis. Specifically, the conditional power for an analysis on the primary efficacy outcome based on 40% of the randomized population (n = 267) with follow-up to 3 months was <30% in both treatment arms. The committee noted that there were no safety concerns. By that time, 1,527 patients undergoing PPCI for acute STEMI had been screened (Figure 1) and 440 patients (mean age 60.5 years, 85% male) had been randomized (151 placebo, 144 alteplase 10 mg, 145 alteplase 20 mg) (Table 1 and Supplemental Table 1). The distribution of randomized participants by ischemic time was as follows: <2 h, 107 (24.3%); ≥2 but <4 h, 235 (53.4%); ≥4 to 6 h, 98 (22.3%). All of the randomized participants were included in those analyses for which they had data available. Seventeen patients (3.9%) withdrew from the study during follow-up.

**Table 1:** Baseline Clinical Characteristics, Ischemic Time of the Randomized Participants (n = 440)

	<2 h (n = 151)	≥2 But <4 h (n = 144)	≥4 to 6 h (n = 145)	p Value
Clinical				
Age, yrs	58.8 ± 8.8	61.8 ± 10.8	59.5 ± 10.6	0.027∗
Male	97 (90.7)	195 (83.0)	82 (83.7)	0.160†
Race, white	97 (90.7)	219 (93.2)	97 (99.0)	0.023†
Body mass index, kg/m^2^	28.4 ± 5.3	28.5 ± 4.8	27.8 ± 4.4	0.704∗
Presenting characteristics				
Heart rate, beats/min	72.8 ± 25.0	71.6 ± 15.9	76.1 ± 17.6	0.136∗
Systolic blood pressure, mm Hg	128 ± 24	134 ± 26	138 ± 25	0.026∗
Diastolic blood pressure, mm Hg	79 ± 15	80 ± 16	83 ± 16	0.146∗
Infarct location				
Anterior	54 (50.5)	103 (43.8)	34 (34.7)	0.073†
Inferior	46 (43.0)	107 (45.5)	54 (55.1)	0.181†
Lateral	3 (2.8)	0 (0.0)	0 (0.0)	0.025†
Posterior	4 (3.7)	21 (8.9)	8 (8.2)	0.223†
Other	0 (0.0)	4 (1.7)	2 (2.0)	0.422†
Medical history				
Hypertension‡	29 (27.1)	81 (34.5)	31 (31.6)	0.417†
Diabetes mellitus‡	11 (10.3)	34 (14.5)	11 (11.2)	0.565†
Hypercholesterolemia‡	22 (20.6)	59 (25.1)	21 (21.4)	0.615†
Smoking‡				
Current	52 (48.6)	106 (45.1)	51 (52.0)	0.490†
Former, stopped >3 months	14 (13.1)	51 (21.7)	19 (19.4)	0.168†
Never	41 (38.3)	78 (33.2)	28 (28.6)	0.344†
Percutaneous coronary intervention	4 (3.7)	9 (3.8)	7 (7.1)	0.401†
Coronary artery bypass graft surgery	0 (0.0)	0 (0.0)	0 (0.0)	–
Angina	0 (0.0)	13 (5.5)	4 (4.1)	0.024†
Myocardial infarction	3 (2.8)	12 (5.1)	5 (5.1)	0.652†
Stroke or transient ischemic attack‡	2 (1.9)	2 (0.9)	1 (1.0)	0.833†
Peripheral vascular disease‡	1 (0.9)	8 (3.4)	3 (3.1)	0.459†
Pre-existing maintenance medication				
Aspirin	14 (13.1)	37 (15.7)	15 (15.3)	0.848†
P2Y_12_ inhibitor				
Clopidogrel	1 (0.9)	0 (0.0)	1 (1.0)	0.217†
Ticagrelor or prasugrel	6 (5.6)	11 (4.7)	3 (3.1)	0.750†
Glycoprotein IIb/IIIa inhibitor	27 (25.7)	30 (12.9)	16 (17.6)	0.017†
Statin	23 (21.5)	52 (22.1)	22 (22.4)	1.0†
Beta-blocker	6 (5.6)	20 (8.5)	16 (16.3)	0.030†
ACE inhibitor or ARB	15 (14.0)	46 (19.6)	17 (17.3)	0.457†
Mineralocorticoid receptor antagonist	3 (2.8)	1 (0.4)	0 (0.0)	0.10†
Symptom onset to arrival at PPCI center, h:min	1:16 (0:59–1:26)	2:12 (1:53–2:44)	4:20 (3:51–4:58)	<0.001§
Arrival at PPCI center to reperfusion, min	22 (17–32)	25 (20–36)	26 (19–38)	0.054§
Symptom onset to reperfusion, h:min	1:42 (1:28–1:52)	2:44 (2:22–3:15)	4:47 (4:21–5:31)	<0.001§
Initial blood results on admission				
Hemoglobin, g/l	145.8 ± 12.1	145.1 ± 14.2	146.0 ± 12.3	0.795∗
Platelet count, ×10^9^ l	260 ± 55	259 ± 61	265 ± 73	0.777∗
Creatinine, μmol/l	83.8 ± 17.5	82.0 ± 20.4	75.6 ± 16.0	0.012∗
Troponin, ng/l	37 (18–69)	57 (29–102)	129 (66–246)	<0.001§

Values are mean ± SD, n (%), or median (interquartile range).

ACE = angiotensin-converting enzyme; ARB = angiotensin receptor blocker; IQR = interquartile range; PPCI = primary percutaneous coronary intervention.

∗ The p value was derived using analysis of variance.

† The p value was derived using Fisher exact test.

‡ At least 1 risk factor for coronary artery disease was required for eligibility. Diabetes mellitus was defined as a history of diet-controlled or treated diabetes.

§ The p value was derived using Kruskal-Wallis test.

### Study intervention

Adjunctive study treatment was administered to 435 patients (98.9%); 5 patients did not receive any drug (Figure 1). Two patients (1 randomized to placebo and 1 randomized to 10 mg alteplase) received 20 mg alteplase because an incorrect treatment pack had been selected.

### Primary and secondary outcomes

CMR was performed in 400 patients (90.9%) at 2 to 7 days and 367 patients (83.4%) at 3 months. The median (interquartile range [IQR]) times to CMR at these time points were 4 days (IQR: 3 to 6 days) and 91 days (IQR: 86 to 97 days), respectively.

### Primary outcome

The mean MVO (percentage of left ventricular mass) revealed by CMR 2 to 7 days post-STEMI (primary outcome) was 2.32 ± 4.31% in the placebo group, 2.61 ± 4.49% in the 10 mg alteplase group, and 3.48 ± 5.83% in the 20 mg alteplase group. A linear regression analysis of square root–transformed MVO found no evidence of a treatment effect (mean increase in square root–transformed MVO per 10-mg increase in alteplase dose: 0.15; 95% confidence interval [CI]: −0.12 to 0.42; p = 0.28) (16).

There was a significant interaction between ischemic time and randomized treatment with respect to the primary outcome (mean increase in square root–transformed MVO per 10-mg increase in alteplase dose: 0.56 (95% CI: 0.21 to 0.91; p = 0.009) (Table 2). There was no evidence of a treatment effect on the extent of MVO at 2 to 7 days for patients with ischemic times <4 h. In those with ischemic times of 4 h or more, the amount of MVO (mean percentage of left ventricular mass) at 2 to 7 days increased from 1.14% in those treated with placebo to 3.11% (10 mg) and 5.20% (20 mg) in those treated with alteplase (Central Illustration). Similar results were observed when analyzing treatment as a 3-level or 2-level categorical variable (Table 3).

**Table 2:** Pre-Specified Analyses of the Primary and Secondary Outcomes, Adjusting for Location of MI, by Subgroups of Ischemic Time

		Randomized Treatment Group			Treatment Effect (Trend per 10-mg Dose Increase)	
	n (Missing)	Placebo (n = 151)	Alteplase 10 mg (n = 144)	Alteplase 20 mg (n = 145)	Estimate (95% CI), p Value	Interaction p Value
**Primary Outcome: Extent of MVO (% of LV Mass) at 2–7 Days**						

Summaries of data on original scale (% of LV mass).						
Overall	396 (44)	2.32 ± 4.31	2.61 ± 4.49	3.48 ± 5.83		
Ischemic time						
<2 h	98 (9)	1.35 ± 2.67	1.49 ± 2.71	2.73 ± 5.03		
≥2 but <4 h	215 (20)	3.01 ± 4.99	3.11 ± 5.28	3.16 ± 5.69		
≥4 to 6 h	83 (15)	1.14 ± 2.62	3.11 ± 4.58	5.20 ± 6.86		
Summaries of data on square root–transformed scale, with treatment effect estimates (change in √MVO per 10-mg increase in alteplase dose); estimates reported for all patients, and by subgroups of ischemic time, with interaction test p value.						
Overall	396 (44)	0.91 ± 1.22	0.99 ± 1.28	1.15 ± 1.48	0.12 (−0.04 to 0.28), 0.128	
Ischemic time						0.018
<2 h	98 (9)	0.63 ± 0.99	0.71 ± 1.01	0.93 ± 1.39	0.12 (−0.21 to 0.46), 0.470	
≥2 but <4 h	215 (20)	1.12 ± 1.33	1.10 ± 1.39	1.06 ± 1.44	−0.03 (−0.23 to 0.18), 0.791	
≥4 to 6 h	83 (15)	0.54 ± 0.94	1.14 ± 1.37	1.64 ± 1.61	0.56 (0.21 to 0.91), 0.009	

**Secondary Outcomes**						

MVO present at 2–7 days. Treatment effect reported as odds ratio per 10-mg increase in alteplase dose.						
Overall	396 (44)	59 (43.4)	58 (45.0)	59 (45.0)	1.04 (0.82 to 1.33), 0.733	
Ischemic time						0.076
<2 h	98 (9)	9 (33.3)	16 (40.0)	11 (35.5)	1.01 (0.59 to 1.73), 0.966	
≥2 but <4 h	215 (20)	42 (50.6)	28 (47.5)	32 (43.8)	0.88 (0.64 to 1.20), 0.411	
≥4 to 6 h	85 (15)	8 (30.8)	14 (46.7)	16 (59.3)	1.84 (1.04 to 3.24), 0.036	
Myocardial hemorrhage (% of LV mass) at 2–7 days. Treatment effect reported as mean change per 10-mg increase in alteplase dose.						
Overall	360 (80)	1.56 ± 3.78	1.98 ± 3.68	2.45 ± 4.80	0.46 (−0.005 to 0.97), 0.075	
Ischemic time						0.038
<2 h	90 (17)	0.26 ± 0.71	1.21 ± 2.60	1.37 ± 2.48	0.42 (−0.67 to 1.52), 0.449	
≥2 but <4 h	196 (39)	2.32 ± 4.62	2.34 ± 4.10	2.38 ± 4.92	0.04 (−0.62 to 0.70), 0.903	
≥4 to 6 h	74 (24)	0.48 ± 1.27	2.39 ± 4.08	3.95 ± 6.19	1.74 (0.61 to 2.87), 0.003	
Myocardial hemorrhage present at 2–7 days. Treatment effect reported as odds ratio per 10-mg increase in alteplase dose.						
Overall	378 (62)	52 (40.6)	54 (44.6)	56 (43.4)	1.07 (0.83 to 1.37), 0.603	
Ischemic time						0.044
<2 h	96 (11)	7 (26.9)	15 (38.5)	11 (35.5)	1.16 (0.67 to 2.02), 0.597	
≥2 but <4 h	202 (33)	38 (49.4)	25 (46.3)	29 (40.8)	0.85 (0.61 to 1.18), 0.324	
≥4 to 6 h	80 (18)	7 (28.0)	14 (50.0)	16 (59.3)	1.93 (1.09 to3.45), 0.025	
Infarct size (% of LV mass) at 2–7 days. Data analyzed on original scale; treatment effect reported as relative increase per 10-mg increase in alteplase dose.						
Overall	396 (44)	26.3 ± 13.7	27.3 ± 12.4	26.7 ± 13.4	0.19 (−1.23 to 1.62), 0.7921	
Ischemic time						0.527
<2 h	98 (9)	22.9 ± 15.4	25.9 ± 13.5	24.3 ± 15.0	−0.18 (−3.25 to 2.89), 0.908	
≥2 but <4 h	215 (20)	28.0 ± 13.9	27.3 ± 11.9	27.3 ± 13.5	−0.23 (−2.10 to 1.63), 0.807	
≥4 to 6 h	83 (15)	24.5 ± 10.6	29.1 ± 12.1	27.6 ± 11.5	1.85 (−1.36 to 5.05), 0.258	
LV ejection fraction at 2–7 days. Treatment effect reported as mean change per 10-mg increase in alteplase dose.						
Overall	400 (40)	44.5 ± 8.8	43.6 ± 8.1	44.2 ± 8.4	−0.2 (−1.1 to 0.8), 0.748	
Ischemic time						0.105
<2 h	99 (8)	45.2 ± 8.3	45.1 ± 7.3	45.2 ± 7.1	0.4 (−1.6 to 2.5), 0.664	
≥2 but <4 h	216 (19)	43.4 ± 9.5	44.2 ± 8.0	44.2 ± 8.8	0.3 (−0.9 to 1.5), 0.617	
≥4 to 6 h	85 (13)	47.0 ± 6.2	40.7 ± 8.8	42.9 ± 8.7	−2.2 (−4.3 to −0.1), 0.041	
LV end-systolic volume at 2–7 days. Data analyzed on a logarithmic scale; treatment effect reported as relative increase per 10-mg increase in alteplase dose.						
Overall	400 (40)	95.8 ± 29.8	104.1 ± 33.0	96.6 ± 30.8	1.00 (0.97 to 1.04), 0.897	
Ischemic time						0.277
<2 h	99 (8)	87.3 ± 23.7	102.0 ± 29.2	96.1 ± 30.4	1.03 (0.95 to 1.11), 0.470	
≥2 but <4 h	216 (19)	100.5 ± 32.4	101.2 ± 34.8	95.9 ± 31.1	0.98 (0.93 to 1.02), 0.359	
≥4 to 6 h	85 (13)	90.2 ± 24.2	112.1 ± 33.6	99.2 ± 31.3	1.05 (0.97 to 1.13), 0.269	
LV end-diastolic volume at 2–7 days. Data analyzed on a logarithmic scale; treatment effect reported as relative increase per 10-mg increase in alteplase dose.						
Overall	400 (40)	171.1 ± 36.5	182.5 ± 40.8	171.4 ± 40.1	1.00 (0.97 to 1.03), 0.960	
Ischemic time						0.332
<2 h	99 (8)	160.1 ± 38.8	183.8 ± 36.2	173.7 ± 43.4	1.03 (0.98 to 1.10), 0.245	
≥2 but <4 h	216 (19)	175.5 ± 35.6	178.9 ± 43.0	170.3 ± 39.3	0.98 (0.95 to 1.02), 0.366	
≥4 to 6 h	85 (13)	169.0 ± 35.7	187.4 ± 42.6	171.8 ± 39.5	1.01 (0.95 to 1.07), 0.831	
Myocardial salvage (% LV) at 2–7 days. Data analyzed on original scale; treatment effect reported as relative increase per 10-mg increase in alteplase dose.						
Overall	396 (44)	14.12 ± 9.17	14.60 ± 9.78	14.35 ± 10.17	0.04 (−1.10 to 1.17), 0.948	
Ischemic time						0.125
<2 h	98 (9)	17.84 ± 11.16	15.32 ± 8.48	20.16 ± 11.11	1.09 (−1.35 to 3.52), 0.382	
≥2 but <4 h	215 (20)	12.75 ± 8.16	14.71 ± 10.13	13.51 ± 9.14	0.43 (−1.05 to 1.92), 0.566	
≥4 to 6 h	83 (15)	14.62 ± 9.22	13.41 ± 10.88	9.96 ± 9.00	−2.27 (−4.81 to 0.27), 0.080	
Area under the troponin T (mg/l) curve, 0–24 h. Data analyzed on a logarithmic scale; treatment effect reported as ratios per 10-mg increase in alteplase dose.						
Overall	317 (123)	4.54 ± 5.58	5.94 ± 7.53	5.84 ± 6.23	1.25 (1.07 to 1.46), 0.006	
Ischemic time						0.191
<2 h	85 (22)	3.40 ± 5.62	3.63 ± 3.96	5.27 ± 5.53	1.41 (1.02 to 1.95), 0.036	
≥2 but <4 h	163 (72)	5.42 ± 5.96	6.23 ± 7.07	5.59 ± 5.50	1.10 (0.90 to 1.36), 0.353	
≥4 to 6 h	69 (29)	3.09 ± 3.44	8.43 ± 10.46	7.17 ± 8.53	1.54 (1.08 to 2.20), 0.016	

Values are mean ± SD or n (%), unless otherwise indicated. All outcomes were pre-specified. Treatment effect estimates derived from linear or logistic regression models, modelling the treatment effect as a linear trend across alteplase dose groups (0 mg vs. 10 mg vs. 20 mg). Interaction test p values reported from regression models with ischemic time included as a 3-level categorical variable and interaction with treatment effect. Treatment effect estimates and tests of interaction are based on models assuming a linear trend with alteplase dose. The p values and 95% CI have not been adjusted for multiplicity, therefore these analyses should be interpreted as exploratory and not definitive.

CI = confidence interval; LV = left ventricular; MI = myocardial infarction; MVO = microvascular obstruction.

**Central Illustration:**
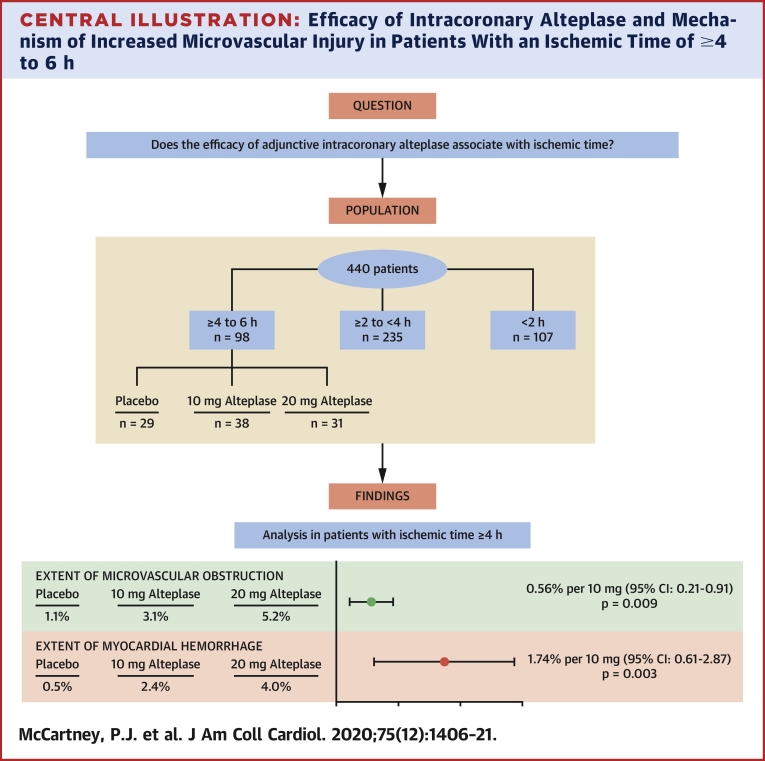
Efficacy of Intracoronary Alteplase and Mechanism of Increased Microvascular Injury in Patients With an Ischemic Time of ≥4 to 6 h The flow diagram groups participants by ischemic time into 3 categories (≥4 to 6 h, n = 98; ≥2 but <4 h, n = 235; <2 h, n = 107), those with an ischemic time of 4 h or more are subgrouped according to treatment group allocation (placebo, n = 29; 10 mg alteplase n = 38; 20 mg alteplase, n = 31). The effect of intracoronary alteplase on the extent of microvascular obstruction and myocardial hemorrhage is shown, including the effect estimates. The estimated mean difference on a square root scale is shown for the extent of microvascular obstruction and the estimated mean difference for myocardial hemorrhage. There was a statistically significant increase in microvascular obstruction and myocardial hemorrhage extent in those patients receiving alteplase. CI = confidence interval.

**Table 3:** Pre-Specified Analyses of the Primary and Secondary Outcomes, Adjusting for Location of MI, by Subgroups of Ischemic Time and Interactions With Treatment Group, Effect Estimates, and Interactions

	Treatment Effect (Alteplase 20 mg vs. Alteplase 10 mg vs. Placebo)			Treatment Effect (Alteplase vs. Placebo)	
	10 mg vs. Placebo Estimate (95% CI), p Value	20 mg vs. Placebo Estimate (95% CI), p Value	Interaction p Value	Estimate (95% CI), p Value	Interaction p Value
**Primary Outcome**					

Extent of MVO (% of LV mass) at 2–7 days. Treatment effects reported as mean differences in square root–transformed MVO between treatment groups (each dose vs. placebo separately, and both active treatment groups combined vs. placebo).					
Overall	0.11 (−0.21 to 0.43), 0.511	0.24 (−0.07 to 0.56), 0.128		0.18 (−0.10 to 0.45), 0.204	
Ischemic time			0.090		0.061
<2 h	0.09 (−0.55 to 0.73), 0.783	0.25 (−0.43 to 0.92), 0.476		0.16 (−0.42 to 0.74), 0.592	
≥2 but <4 h	−0.01 (−0.45 to 0.42), 0.947	−0.06 (−0.47 to 0.35), 0.790		−0.04 (−0.40 to 0.32), 0.837	
≥4 to 6 h	0.53 (−0.15 to 1.22), 0.128	1.12 (0.42 to 1.82), 0.002		0.81 (0.21 to 1.42), 0.009	

**Secondary Outcomes**					

MVO present at 2–7 days. Treatment effects reported as odds ratios between groups.					
Overall	0.12 (0.68 to 1.84), 0.651	1.09 (0.67 to 1.77), 0.734		1.10 (0.72 to 1.69), 0.647	
Ischemic time			0.240		0.147
<2 h	1.35 (0.49 to 3.78), 0.561	1.04 (0.35 to 3.11), 0.940		1.21 (0.47 to 3.09), 0.689	
≥2 but <4 h	0.89 (0.45 to 1.74), 0.726	0.77 (0.41 to 1.45), 0.410		0.82 (0.47 to 1.42), 0.476	
≥4 to 6 h	1.86 (0.61 to 5.61), 0.272	3.38 (1.08 to 10.55), 0.036		2.46 (0.92 to 6.60), 0.073	
Myocardial hemorrhage (% of LV mass) at 2–7 days. Treatment effects reported as mean differences between groups.					
Overall	0.55 (−0.50 to 1.60), 0.304	0.93 (−0.09 to 1.94), 0.074		0.75 (−0.14 to 1.65), 0.100	
Ischemic time			0.149		0.097
<2 h	0.94 (−1.16 to 3.05), 0.380	0.90 (−1.31 to 3.10), 0.425		0.93 (−0.99 to 2.85), 0.343	
≥2 but <4 h	0.00 (−1.43 to 1.44), 0.996	0.08 (−1.24 to 1.41), 0.903		0.05 (−1.12 to 1.22), 0.935	
≥4 to 6 h	1.63 (−0.64 to 3.91), 0.160	3.49 (1.22 to 5.75), 0.003		2.57 (0.59 to 4.54), 0.011	
Myocardial hemorrhage present at 2–7 days. Treatment effects reported as odds ratios between groups.					
Overall	1.25 (0.75 to 2.08), 0.401	1.14 (0.69 to 1.89), 0.598		1.19 (0.77 to 1.85), 0.434	
Ischemic time			0.150		0.059
<2 h	1.72 (0.58 to 5.09), 0.327	1.41 (0.45 to 4.43), 0.554		1.58 (0.58 to 4.28), 0.369	
≥2 but <4 h	0.89 (0.44 to 1.80), 0.748	0.72 (0.37 to 1.38), 0.322		0.79 (0.45 to 1.40), 0.418	
≥4 to 6 h	2.42 (0.76 to 7.64), 0.133	3.81 (1.19 to 12.25), 0.025		3.02 (1.08 to 8.42), 0.035	
Infarct size (% of LV mass) at 2–7 days. Data analyzed on original scale; treatment effect reported as relative increase per 10-mg increase in alteplase dose.					
Overall	1.23 (−1.66 to 4.11), 0.404	0.38 (−2.48 to 3.23), 0.795		0.79 (−1.68 to 3.27), 0.530	
Ischemic time			0.600		0.475
<2 h	3.36 (−2.45 to 9.16), 0.258	−0.17 (−6.32 to 5.97), 0.956		1.82 (−3.44 to 7.08), 0.498	
≥2 but <4 h	−0.47 (−4.44 to 3.50), 0.818	−0.46 (−4.20 to 3.28), 0.810		−0.46 (−3.73 to 2.80), 0.780	
≥4 to 6 h	2.85 (−3.41 to 9.11), 0.373	3.71 (−2.70 to 10.12), 0.257		3.26 (−2.25 to 8.77), 0.246	
LV ejection fraction at 2–7 days. Treatment effects reported as mean differences between groups.					
Overall	−0.9 (−2.8 to 1.0), 0.367	−0.3 (−2.2 to 1.6), 0.752		−0.6 (−2.2 to 1.1), 0.483	
Ischemic time			0.117		0.027
<2 h	−0.2 (−4.0 to 3.6), 0.915	0.9 (−3.2 to 4.9), 0.679		0.3 (−3.2 to 3.7), 0.884	
≥2 but <4 h	0.7 (−2.0 to 3.3), 0.616	0.6 (−1.8 to 3.1), 0.621		0.6 (−1.5 to 2.8), 0.557	
≥4 to 6 h	−5.5 (−9.5 to −1.4), 0.008	−4.5 (−8.7 to −0.2), 0.039		−5.0 (−8.6 to −1.4), 0.007	
LV end-systolic volume at 2–7 days. Data analyzed on a logarithmic scale; treatment effects reported as relative differences between groups.					
Overall	1.08 (1.01 to 1.16), 0.027	1.00 (0.94 to 1.08), 0.907		1.04 (0.98 to 1.11), 0.184	
Ischemic time			0.222		0.053
<2 h	1.17 (1.01 to 1.34), 0.032	1.06 (0.92 to 1.23), 0.424		1.12 (0.99 to 1.27), 0.082	
≥2 but <4 h	1.00 (0.91 to 1.11), 0.943	0.96 (0.87 to 1.05), 0.346		0.98 (0.90 to 1.06), 0.576	
≥4 to 6 h	1.21 (1.04 to 1.40), 0.016	1.10 (0.94 to 1.28), 0.255		1.15 (1.01 to 1.32), 0.038	
LV end-diastolic volume at 2–7 days. Data analyzed on a logarithmic scale; treatment effects reported as relative differences between groups.					
Overall	1.06 (1.01 to 1.12), 0.947	1.00 (0.95 to 1.05), 0.947		1.03 (0.98 to 1.08), 0.210	
Ischemic time			0.314		0.081
<2 h	1.16 (1.04 to 1.29), 0.007	1.08 (0.96 to 1.21), 0.211		1.12 (1.02 to 1.24), 0.021	
≥2 but <4 h	1.01 (0.94 to 1.09), 0.743	0.97 (0.90 to 1.04), 0.349		0.99 (0.93 to 1.05), 0.676	
≥4 to 6 h	1.10 (0.98 to 1.23), 0.117	1.01 (0.90 to 1.14), 0.814		1.06 (0.95 to 1.17), 0.282	
Myocardial salvage (% LV) at 2–7 days. Data analyzed on original scale; treatment effect reported as relative increase per 10-mg increase in alteplase dose.					
Overall	0.04 (−2.26 to 2.34), 0.975	0.08 (−2.20 to 2.35), 0.948		0.06 (−1.92 to 2.03), 0.955	
Ischemic time			0.071		0.337
<2 h	−2.44 (−7.03 to 2.16), 0.299	1.99 (−2.87 to 6.86), 0.422		−0.51 (−4.70 to 3.68), 0.811	
≥2 but <4 h	2.00 (−1.14 to 5.14), 0.212	0.81 (−2.15 to 3.77), 0.592		1.35 (−1.25 to 3.94), 0.309	
≥4 to 6 h	−1.60 (−6.55 to 3.35), 0.527	−4.54 (−9.61 to 0.53), 0.080		−3.00 (−7.39 to 1.38), 0.179	
Area under the troponin T (mg/l) curve, 0–24 h. Data analyzed on a logarithmic scale; treatment effects reported as relative differences between groups.					
Overall	1.61 (1.17 to 2.22), 0.003	1.56 (1.14 to 2.13), 0.006		1.58 (1.21 to 2.08), 0.001	
Ischemic time			0.257		0.081
<2 h	1.76 (0.96 to 3.21), 0.067	2.00 (1.05 to 3.79), 0.034		1.86 (1.08 to 3.19), 0.025	
≥2 but <4 h	1.24 (0.78 to 1.96), 0.362	1.21 (0.80 to 1.83), 0.363		1.22 (0.85 to 1.76), 0.278	
≥4 to 6 h	2.82 (1.44 to 5.54), 0.003	2.44 (1.20 to 4.94), 0.014		2.64 (1.44 to 4.83), 0.002	

All outcomes were pre-specified. Treatment effect estimates derived from linear or logistic regression models, modelling the treatment effect as a 3-level categorical variable or as a 2-level categorical variable (active vs. placebo). Interaction test p values reported from regression models with ischemic time included as a 3-level categorical variable and interaction with treatment effect. P values and 95% CI presented in this table have not been adjusted for multiplicity, therefore these analyses should be interpreted as exploratory and not definitive.

Abbreviations as in Table 2.

### Secondary outcomes

#### CMR parameters at 2 to 7 days

Although the interaction between ischemic time and treatment in relation to the binary outcome of the presence of any MVO did not reach statistical significance (odds ratio [OR]: 1.84; 95% CI: 1.04 to 3.24; p = 0.036; interaction p = 0.076), there was a trend toward a higher prevalence with increasing dose in patients presenting ≥4 to 6 h (Table 2), but no evidence of a treatment effect with shorter ischemic times. A similar pattern was observed regarding myocardial hemorrhage, with an increasing prevalence in those with ischemic times ≥4 to 6 h (OR per 10-mg increase in alteplase dose: 1.93; 95% CI: 1.09 to 3.45; p = 0.025), but no significant trend with shorter ischemic times (p value for interaction = 0.044), as well as an increasing extent of myocardial hemorrhage (percentage of left ventricular mass) in those with longer ischemic times (1.74% increase per 10-mg increase in alteplase dose; 95% CI: 0.61 to 2.87; p = 0.003) (Central Illustration), but no evidence of treatment effects with shorter ischemic times (p for interaction = 0.038). The statistical evidence for interactions was weaker when considering treatment effects categorically (Table 3), but the general pattern of associations was very similar, with poorer outcomes in those treated with alteplase (particularly the 20-mg dose) when the ischemic time was ≥4 to 6 h. Similar trends were observed when patients were categorized by the location of MI, anterior and nonanterior (Table 4).

**Table 4:** Pre-Specified Analyses of the Primary and Selected Secondary Outcomes, Adjusting for Location of MI, by Subgroups of Ischemic Time and MI Location (Anterior/Nonanterior)

		Randomized Treatment Group			Treatment Effect (Trend per 10-mg Dose Increase)	
	n (Missing)	Placebo (n = 151)	Alteplase 10 mg (n = 144)	Alteplase 20 mg (n = 145)	Estimate (95% CI), p Value	Interaction p Value
**Primary Outcome**						

Extent of MVO (% of LV mass) at 2–7 days. Summaries of data on square root–transformed scale, with treatment effect estimates (change in √MVO per 10-mg increase in alteplase dose); estimates reported for all patients, and by subgroups of ischemic time, with interaction test p value.						
Anterior MI						
Overall	178 (15)	1.16 ± 1.44	1.08 ± 1.28	1.42 ± 1.64	0.15 (−0.11 to 0.42), 0.249	
Ischemic time						0.264
<2 h	50 (4)	0.81 ± 1.16	0.77 ± 0.98	1.16 ± 1.51	0.19 (−0.32 to 0.70), 0.458	
≥2 but <4 h	96 (9)	1.37 ± 1.55	1.28 ± 1.40	1.41 ± 1.61	0.02 (−0.33 to 0.36), 0.929	
≥4 to 6 h	32 (2)	0.77 ± 1.26	1.11 ± 1.37	2.10 ± 2.06	0.65 (−0.04 to 1.34), 0.064	
Nonanterior MI						
Overall	218 (29)	0.72 ± 0.99	0.91 ± 1.29	0.92 ± 1.29	0.10 (−0.09 to 0.29), 0.316	
Ischemic time						0.049
<2 h	48 (5)	0.47 ± 0.81	0.66 ± 1.04	0.56 ± 1.12	0.05 (−0.40 to 0.51), 0.823	
≥2 but <4 h	119 (11)	0.91 ± 1.09	0.96 ± 1.39	0.78 ± 1.24	-0.06 (−0.31 to 0.19), 0.630	
≥4 to 6 h	51 (13)	0.42 ± 0.74	1.16 ± 1.42	1.45 ± 1.41	0.51 (0.13 to 0.90), 0.009	

**Secondary Outcomes**						

MVO present at 2–7 days. Treatment effect reported as odds ratio per 10-mg increase in alteplase dose.						
Anterior MI						
Overall	178 (15)	28 (46.7)	29 (49.2)	31 (52.5)	1.16 (0.80 to 1.66), 0.437	
Ischemic time						0.613
<2 h	50 (4)	5 ± 38.5	8 ± 44.4	8 ± 42.1	1.07 (0.52 to 2.17), 0.862	
≥2 but <4 h	96 (9)	20 ± 52.6	14 ± 53.8	18 ± 56.2	1.08 (0.67 to 1.72), 0.764	
≥4 to 6 h	32 (2)	3 ± 33.3	7 ± 46.7	5 ± 62.5	1.82 (0.67 to 4.94), 0.237	
Nonanterior MI						
Overall	218 (29)	31 (40.8)	29 (41.4)	28 (38.9)	0.96 (0.69 to 1.33), 0.788	
Ischemic time						0.089
<2 h	48 (5)	4 ± 28.6	8 ± 36.4	3 ± 25.0	0.93 (0.41 to 2.15), 0.874	
≥2 but <4 h	119 (11)	22 ± 48.9	14 ± 42.4	14 ± 34.1	0.74 (0.48 to 1.14), 0.169	
≥4 to 6 h	51 (13)	5 ± 29.4	7 ± 46.7	11 ± 57.9	1.81 (0.91 to 3.59), 0.092	
Myocardial hemorrhage (% of LV mass) at 2–7 days. Treatment effect reported as mean change per 10-mg increase in alteplase dose.						
Anterior MI						
Overall	148 (35)	2.29 ± 5.13	2.22 ± 3.54	3.41 ± 5.81	0.69 (−0.25 to 1.63), 0.148	
Ischemic time						0.079
<2 h	44 (10)	0.38 ± 0.85	1.43 ± 2.94	1.86 ± 2.72	0.71 (−1.13 to 2.54), 0.450	
≥2 but <4 h	87 (18)	3.30 ± 6.07	2.89 ± 4.07	3.52 ± 6.01	0.10 (−1.09 to 1.29), 0.867	
≥4 to 6 h	27 (7)	0.26 ± 0.50	1.94 ± 3.14	6.89 ± 9.33	3.32 (0.79 to 5.84), 0.010	
Nonanterior MI						
Overall	202 (45)	1.05 ± 2.34	1.78 ± 3.82	1.70 ± 3.70	0.32 (−0.23 to 0.86), 0.255	
Ischemic time						0.250
<2 h	46 (7)	0.18 ± 0.61	1.04 ± 2.38	0.63 ± 1.95	0.24 (−1.06 to 1.53), 0.721	
≥2 but <4 h	109 (21)	1.52 ± 2.84	1.87 ± 4.14	1.52 ± 3.76	0.00 (−0.72 to 0.72), 1.000	
≥4 to 6 h	47 (17)	0.57 ± 1.49	2.88 ± 5.01	2.80 ± 4.29	1.11 (0.01 to 2.20), 0.047	
Myocardial hemorrhage present at 2–7 days. Treatment effect reported as odds ratio per 10-mg increase in alteplase dose.						
Anterior MI						
Overall	168 (25)	25 (44.6)	27 (49.1)	28 (49.1)	1.12 (0.77 to 1.62), 0.558	
Ischemic time						0.661
<2 h	48 (6)	4 ± 33.3	8 ± 47.1	8 ± 42.1	1.16 (0.56 to 2.41), 0.689	
≥2 but <4 h	90 (15)	18 ± 50.0	12 ± 50.0	15 ± 50.0	1.00 (0.62 to 1.62), 1.000	
≥4 to 6 h	30 (4)	3 ± 37.5	7 ± 50.0	5 ± 62.5	1.67 (0.61 to 4.59), 0.323	
Nonanterior MI						
Overall	210 (37)	27 (37.5)	27 (40.9)	28 (38.9)	1.02 (0.73 to 1.43), 0.910	
Ischemic time						0.045
<2 h	48 (5)	3 ± 21.4	7 (31.8)	3 ± 25.0	1.11 (0.47 to 2.64), 0.811	
≥2 but <4 h	112 (18)	20 ± 48.8	13 (43.3)	14 ± 34.1	0.74 (0.47 to 1.15), 0.181	
≥4 to 6 h	50 (14)	4 ± 23.5	7 (50.0)	14 ± 34.1	2.06 (1.02 to 4.18), 0.044	
Infarct size (% of LV mass) at 2–7 days. Data analyzed on original scale; treatment effect reported as relative increase per 10-mg increase in alteplase dose.						
Anterior MI						
Overall	178 (15)	33.1 ± 14.3	33.8 ± 11.9	31.9 ± 15.3	−0.40 (−2.90 to 2.10), 0.756	
Ischemic time						0.453
<2 h	50 (8)	27.5 ± 17.0	34.5 ± 13.8	28.1 ± 16.4	−0.16 (−5.01 to 4.70), 0.950	
≥2 but <4 h	96 (19)	36.1 ± 13.6	32.6 ± 12.1	33.4 ± 14.7	−1.41 (−4.66 to 1.85), 0.3974	
≥4 to 6 h	32 (13)	28.7 ± 10.3	35.1 ± 9.5	35.1 ± 14.9	3.30 (−3.30 to 9.89), 0.327	
Nonanterior MI						
Overall	218 (29)	20.9 ± 10.6	21.8 ± 10.0	22.3 ± 9.9	0.65 (−0.97 to 2.27), 0.431	
Ischemic time						0.874
<2 h	48 (5)	18.7 ± 12.8	18.9 ± 8.2	18.2 ± 10.5	−0.22 (−4.11 to 3.67), 0.912	
≥2 but <4 h	119 (11)	21.1 ± 10.1	23.2 ± 10.1	22.6 ± 10.3	0.73 (−1.41 to 2.86), 0.505	
≥4 to 6 h	51 (13)	22.3 ± 10.3	23.2 ± 11.7	24.5 ± 8.2	1.10 (−2.20 to 4.40), 0.513	

Values are mean ± SD or n (%), unless otherwise indicated. All outcomes were pre-specified. Treatment effect estimates derived from linear or logistic regression models, modelling the treatment effect as a linear trend across alteplase dose groups (0 mg vs. 10 mg vs. 20 mg). Interaction test p values reported from regression models with ischemic time included as a 3-level categorical variable and interaction with treatment effect. Treatment effect estimates and tests of interaction are based on models assuming a linear trend with alteplase dose. The p values and 95% CI have not been adjusted for multiplicity, therefore these analyses should be interpreted as exploratory and not definitive.

Abbreviations as in Table 2.

Left ventricular ejection fraction 2 to 7 days post-STEMI was lower in patients presenting ≥4 to 6 h who were treated with alteplase (10 mg or 20 mg) compared with in those who received placebo (mean difference: −5.0%; 95% CI: −8.6% to −1.4%; p = 0.007) (Table 3), with no evidence of treatment effects (active vs. placebo) with shorter ischemic times (interaction p value = 0.027). The interaction with ischemic time was not statistically significant when treatment was assessed as a 3-level categorical variable (Table 3) or as a trend across treatment groups (Table 2), though the treatment effect estimates demonstrated a similar pattern. No significant interactions were observed between ischemic time and treatment for left ventricular end-systolic or end-diastolic volumes, regardless of how the treatment effect was modelled. Patterns of treatment effects in relation to left ventricular measures at 3 months were similar, though with fewer statistically significant associations (Supplemental Table 2). There was no evidence of any treatment effects in relation to infarct size, or myocardial salvage index at 2 to 7 days or 3 months.

### Blood chemistry

The AUC for troponin T (ng/l) measured at baseline and 2 and 24 h post-reperfusion in 317 subjects was increased in both treatment groups compared with the placebo group, for those treated with alteplase, the relative difference was 1.53 (95% CI: 1.12 to 2.11; p = 0.008) (16). Troponin T AUC was 35% higher in patients treated with 20 mg of alteplase versus placebo. There was no interaction among troponin T AUC, ischemic time, and treatment with alteplase compared with placebo (Table 2).

### Hematology and coagulation, 2-h time point

By 2 h after study drug administration, circulating concentrations of fibrin D-dimers were increased in the alteplase groups compared with in the placebo group (Supplemental Table 3). There were no statistically significant interactions observed for fibrin D-dimers, prothrombin F1 + 2 (a measure of thrombin activation), tissue plasminogen activator (a measure of endogenous tissue plasminogen activator and any circulating alteplase), plasminogen, or fibrinogen (Supplemental Table 4).

## Discussion

The principal findings from the T-TIME trial were that the intervention was feasible but not effective (16). Adjunctive, low-dose intracoronary alteplase administered after coronary reperfusion and before stent implantation did not reduce the amount of MVO revealed by cardiac CMR 2 to 7 days post-STEMI.

**Figure 2:**
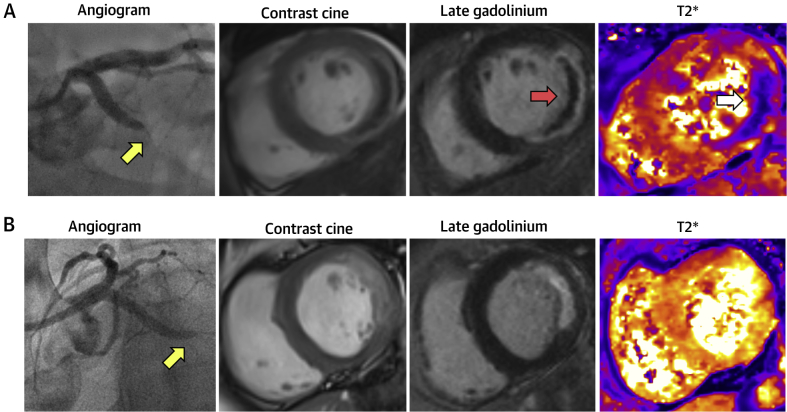
Clinical Case Examples Two patients, both with acute lateral ST-segment elevation myocardial infarction treated successfully with primary percutaneous coronary intervention. Each patient had TIMI (Thrombolysis In Myocardial Infarction) flow grade 0 at initial angiography and TIMI flow grade 3 (normal flow grade) at the end of percutaneous coronary intervention. The first with an ischemic time of 5 h and the second 3 h. Cardiac magnetic resonance (CMR) was performed at 3 days post-reperfusion. **(A)** Patient with hemorrhagic infarction on CMR. Diagnostic coronary angiogram demonstrated an occluded circumflex artery **(yellow arrow)**. T_2_*-CMR **(far right)** revealed myocardial hemorrhage **(white arrow)** within the infarct core. Late gadolinium-enhanced CMR revealed microvascular obstruction **(middle, red arrow)** within the bright area of infarction. The microvascular obstruction within the infarct core spatially corresponded with the myocardial hemorrhage. This represents a case of failed microvascular reperfusion despite successful percutaneous coronary intervention. **(B)** Patient with a lateral infarct but no CMR evidence of reperfusion injury. Diagnostic coronary angiogram demonstrated an occluded circumflex artery **(yellow arrow)**. Late gadolinium-enhanced CMR revealed a lateral infarct with no evidence of microvascular obstruction and no evidence of hemorrhagic transformation on T_2_*-CMR. This represents a case of successful microvascular reperfusion.

In this pre-specified analysis, low-dose intracoronary alteplase administered during PPCI was associated with an increase in the amount of MVO in patients with an ischemic time of 4 h or more. When the interaction test between ischemic time and treatment was performed as a trend across treatment groups, we observed a statistically significant interaction, indicating a dose-dependent increase in MVO with alteplase in association with the duration of ischemia. An increase in the proportion of patients with myocardial hemorrhage as well as an increase in the amount of hemorrhage by ischemic time and treatment with alteplase (10 mg, 20 mg) was observed. These dose effects were driven by those patients receiving 20 mg of alteplase. In the subgroup of patients with the longest ischemic time (≥4 to 6 h), treatment with 20 mg alteplase was also associated with a lower left ventricular ejection fraction at 2 to 7 days. The results do not support this therapeutic approach, especially in those STEMI patients presenting with an ischemic time of 4 h or more, in whom MVO and myocardial hemorrhage may be increased. Clinical case examples are shown in Figure 2. Whether giving low-dose fibrinolysis at the end of PPCI in patients presenting with an ischemic time <4 h might be beneficial merits prospective assessment.

The mechanism for an increase in microvascular injury in patients with an ischemic time ≥4 to 6 h treated with alteplase likely involves hemorrhagic transformation within the infarct core. Prolonged ischemia leads to capillary degradation (26) and myocyte necrosis, and in these circumstances, alteplase appears to promote tissue hemorrhage. Myocardial hemorrhage underpins adverse left ventricular remodeling (27,28) and is independently predictive of an adverse cardiac prognosis in the longer term (27,29). An increase in the extravasation of blood into the interstitial space at the infarct core results in the external compression of the capillary bed with an associated exponential increase in microvascular resistance. This external compressive mass potentiates progression of microvascular damage. Myocardial hemorrhage is a pathological subset of MVO, as revealed by CMR imaging (27), in addition to the effect on microvascular injury, the increase in interstitial mass increases the extent of MVO as measured by CMR due to the associated mass effect.

We observed a close relationship between MVO and myocardial hemorrhage. Myocardial hemorrhage did not occur in the absence of MVO, although hemorrhage was present in the majority of patients with MVO, it was not universal. We found that myocardial hemorrhage occurred in all patients with MVO who presented with a prolonged ischemic time (4 h or more) who then went on to receive alteplase (10-mg or 20-mg dose). This was not the case in those patients receiving placebo or in patients with an ischemic time of <4 h. This increase in the proportion of patients with myocardial hemorrhage versus MVO without myocardial hemorrhage by ischemic time and treatment group highlights the potential deleterious effects of adjunctive alteplase in patients with established microvascular injury. The increase in the extent of myocardial hemorrhage may be explained by the observation that MVO and myocardial hemorrhage are the same phenomenon in the majority of cases. A multicenter cohort study (30) previously reported increases in myocardial hemorrhage in patients receiving periprocedural glycoprotein IIb/IIIa inhibitor and an animal study (31) demonstrated an increased incidence of myocardial hemorrhage with the use of additional glycoprotein IIb/IIIa inhibitors. More aggressive antithrombotic treatment may promote tissue hemorrhage especially in the context of established microvascular injury.

The relationship between vascular permeability post-MI and tissue hemorrhage was highlighted in a study investigating the role of angiopoietin-like protein 2, which has been linked to endothelial cell junction stability and vascular permeability in mice. The investigators demonstrated that angiopoietin-like protein 2 mediates protection against post-ischemic tissue damage through preservation of the endothelial cell tissue barrier with associated reductions in myocardial hemorrhage and infarct size (26).

The detection of myocardial hemorrhage in vivo is limited by difficulty in obtaining reliable diagnostic quality images in a proportion of patients, which for T_2_* imaging typically requires long breath holds with minimal respiratory movement. This is highlighted by the observation that in our study, an assessment of the extent of MVO was possible in 396 of 440 participants compared with 360 of 440 for myocardial hemorrhage. This difference, reflecting a limitation in the diagnostic performance of T_2_* imaging, is comparable to previous reports (32). These results help explain why detection of myocardial hemorrhage may prove challenging, especially in those patients with a limited amount of myocardial hemorrhage. The result provides insights into why some patients may have detectable MVO but no myocardial hemorrhage.

The overall clinical relevance of our findings is highlighted by a trend on ischemic time toward reduced ejection fraction in patients receiving alteplase versus placebo. We provide evidence that increased myocardial hemorrhage is causally related to a reduction in left ventricular function and adverse left ventricular remodeling.

The T-TIME study has several strengths. The primary and secondary outcomes were analyzed using core laboratory methods. The study intervention and source data analyses were conducted in a double-blind manner, minimizing the risk of bias. The design specified multimodality testing including a time-course AUC analysis of the circulating concentrations of troponin T. The coagulation results have been useful to inform the safety of intracoronary alteplase as an adjunct during PPCI.

MVO presents an unmet therapeutic need and there is widespread interest in the potential efficacy of intra-coronary fibrinolytic therapy during PPCI. Two multicenter, international trials are scheduled to investigate the efficacy of reduced doses of either alteplase (STRIVE [Adjunctive, Low-dose tPA in Primary PCI for STEMI]; NCT03335839) or tenecteplase (RESTORE-MI [Restoring Microcirculatory Perfusion in ST-Elevation Myocardial Infarction (STEMI)]; ACTRN12618000778280) (Supplemental Appendix). Considering eligibility criteria in these trials, the ischemic time limit is 12 h. Furthermore, RESTORE-MI selects patients with evidence of microvascular dysfunction (index of microcirculatory resistance >32) in the infarct-related artery at the end of PCI. Our results suggest this risk-based selection strategy may enroll patients at risk of myocardial hemorrhage that, based on our findings, may be exacerbated by intracoronary lytic therapy. The new knowledge from the T-TIME study seems relevant to the design of these trials and to clinicians in practice when considering the use of intracoronary alteplase as a bail-out option in patients with massive thrombosis. Finally, PPCI is not available for many patients due to both geographical and socioeconomic factors (33). As a result, intravenous thrombolysis is the primary reperfusion strategy for many STEMI patients worldwide. Our findings are potentially relevant for these patients. The GUSTO-1 (Global Utilization of t-Pa and Streptokinase for Occluded Coronary Arteries) trial evaluated the effects of intravenous thrombolysis in over 40,000 STEMI patients and those with a symptom onset to treatment time of 4 to 6 h had a >40% relative increase in mortality at 30 days when compared with patients with shorter treatment times (18). Increases in MVO and myocardial hemorrhage in patients with a prolonged ischemic time treated with thrombolysis may be a contributing factor for this increase in mortality. This could be considered by clinicians when a choice is available between prompt thrombolysis and delayed PCI beyond the guideline-directed 120-min target in patients with prolonged ischemic times.

### Study limitations

First, the study was discontinued when pre-specified futility criteria were met. The objectives of this phase 2 trial included evidence synthesis for mechanisms evaluation as well as efficacy. To an extent, premature discontinuation limits mechanism evaluation. Second, although ischemic time was a pre-specified subgroup, no adjustment for multiplicity was made in this subgroup analysis. Finally, the decision to explore treatment effects as trends across treatment groups was made post hoc, this provided stronger evidence of the interaction based on ischemic time and treatment with alteplase. The results of this analysis should therefore be interpreted as exploratory and not definitive.

## Conclusions

In patients presenting with acute STEMI and an ischemic time ≥4 to 6 h, adjunctive, low-dose, intracoronary alteplase given during PPCI may increase MVO and myocardial hemorrhage and reduce left ventricular ejection fraction. The mechanisms may involve hemorrhagic transformation within the infarct core. The results do not support administering intracoronary alteplase in patients with STEMI presenting with an ischemic time ≥4 to 6 h.

Perspectives **COMPETENCY IN PATIENT CARE AND PROCEDURAL SKILLS:** In patients with acute STEMI and an ischemic time ≥4 to 6 h undergoing PPCI, low-dose intracoronary alteplase increases MVO and myocardial hemorrhage and worsens left ventricular function.

**TRANSLATIONAL OUTLOOK:** Future studies of intracoronary thrombolysis should focus on patients presenting within 4 h of symptom onset.
