# Supplementary material for: Low-Dose Alteplase During Primary Percutaneous Coronary Intervention According to Ischemic Time
Source: J Am Coll Cardiol. 2020 Mar 31;75(12):1406–21. doi: 10.1016/j.jacc.2020.01.041 (PMC7109518; doi:10.1016/j.jacc.2020.01.041)
Supplement: Online Data [file mmc1.docx]

# Supplementary Material

## Low-Dose Alteplase During Primary Percutaneous Coronary Intervention According to Ischemic Time

ClinicalTrials.gov: NCT02257294.

# Supplementary Methods

## Participants and eligibility criteria

Patients with a clinical diagnosis of acute STEMI were eligible for randomization according to the following eligibility criteria

### Inclusion

- Acute myocardial infarction (symptom onset ≤ 6 hours) with persistent ST segment elevation or recent left bundle branch block
- Coronary artery occlusion (TIMI coronary flow grade 0 or 1) or, impaired coronary flow (TIMI flow grade 2, slow but complete filling) in the presence of definite angiographic evidence of thrombus (TIMI grade 2+).
- Proximal-mid culprit lesion location in a major coronary artery (i.e. the right, left anterior descending, intermediate or circumflex coronary artery)
- Radial artery access

### Exclusion

Clinical criteria that would exclude the patient from the trial were evaluated by medical, research and nursing staff when the patient arrives in the catheter laboratory.

### Coronary

- Normal flow in the culprit coronary artery at initial angiography (TIMI grade 3)
- Functional coronary collateral supply (Rentrop grade 2/3) to the culprit artery
- Previous infarction in the culprit artery (known or suspected clinically, e.g. wall motion abnormality revealed by echocardiography)

### Clinical

- Cardiogenic shock (Killip Class IV)
- Multivessel PCI intended before cardiac magnetic resonance imaging (MRI) intended for day 2 – 7
- Previous infarction in the culprit artery (known or suspected clinically)
- Body weight estimated to be <60 kg
- Non-cardiac co-morbidity with expected survival <1 year
- Contra-indication to contrast-enhanced MRI

Pacemaker

- Implantable defibrillator
- Known impaired renal function (eGFR<30ml/min)
- Significant bleeding disorder either at present or within the past 6 months
- Patients with current concomitant oral anticoagulant therapy (INR > 1.3), including apixaban, dabigatran, and rivaroxaban
- Any history of central nervous system damage (i.e. neoplasm, aneurysm, intracranial or spinal surgery)
- Known hemorrhagic diathesis
- Severe hypertension (BP >180/110 mmHg) not controlled by medical therapy
- Major surgery, biopsy of a parenchymal organ, or significant trauma within the past 3 months (this includes any trauma associated with the current AMI)
- Recent trauma to the head or cranium (<2 months)
- Prolonged cardiopulmonary resuscitation (>2 minutes) within the past 2 weeks
- Acute pericarditis and/or subacute bacterial endocarditis e.g. valve mass or vegetation revealed by echocardiography
- Acute pancreatitis
- Severe hepatic dysfunction, including hepatic failure, cirrhosis, portal hypertension (esophageal varices) and active hepatitis
- Active peptic ulceration
- Arterial aneurysm and known arterial/venous malformation
- Neoplasm with increased bleeding risk
- Any known history of hemorrhagic stroke or stroke of unknown origin
- Known history of ischemic stroke or transient ischemic attack in the preceding 6 months
- Dementia
- Hypersensitivity to gentamicin or natural rubber
- Incapacity or inability to provide informed consent
- Previous randomization to this study or participation in a study with an investigational drug or medical device within 90 days prior to randomization
- Women of child-bearing potential (i.e. pre-menopause) or breast feeding.
- Requirement for immunosuppressive drug therapy at any time during the past 3 months; whether administered orally, subcutaneously, intramuscularly or intravenously. This would include corticosteroids (but not inhaled or topical), drugs used following transplantation (e.g. tacrolimus, cyclosporine), anti-metabolite therapies (e.g. mycophenolic acid (Myfortic), azathioprine, leflunomide (Arava)), and immunomodulators including biologics (e.g. adalimumab (HUMIRA), etanercept (Enbrel), aldesleukin), and DMARDS (cyclophosphamide, methotrexate, etc.). Please note this list is not exhaustive and a requirement for other immunosuppressive drugs not listed would also exclude the patient.
- Active or prophylactic treatment with oral or parenteral antibiotic, antifungal or antiviral therapy to prevent or treat infection.
- Any anti-cancer treatment (excluding surgery as this is covered above) at any time during the past 3 months including chemotherapy, radiotherapy and treatment with biologics such as Vascular Endothelial Growth Factor Receptor (VEGFR) inhibitors

(e.g. bevacizumab, pazopanib).

- Any significant concurrent or recent condition(s) not listed above that in the opinion of the treating clinician would pose an additional risk for the patient.

## Informed consent

Screening, witnessed verbal informed consent, study drug administration and acute assessments of efficacy took place during the standard of care emergency primary PCI procedure in the cardiac catheterization laboratory. A screening log was prospectively completed. Only patients who were sufficiently well to understand the information about the study, as described by the attending cardiologist, were eligible to participate. The decision as to whether a patient is eligible to be included was made and documented by the cardiologist. The study information sheet that had been approved by the research ethics committee was subsequently provided to each participant on the ward where written informed consent was obtained. The participants were followed-up unless consent was withdrawn.

## Standard care

Standard care for coronary reperfusion was recommended according to contemporary practice guidelines(1) using either balloon angioplasty or aspiration thrombectomy for thrombus-containing lesions. A coronary balloon diameter (mm) vs. lumen diameter (mm) relationship of <1:1 and a low inflation pressure were recommended to minimize thrombus embolization. The balloon angioplasty was intended to stabilize the thrombotic lesion and prevent vessel re-occlusion prior to stent implantation. Anti-thrombotic therapy included oral anti-platelet drugs and intravenous heparin (5000 IU or as per standard practice) at the first medical contact. The target activated clotting time (ACT) was 250s.

## Interventions

After successful reperfusion (TIMI flow grade ≥2) of the infarct-related artery the participants immediately received the allocated intervention. The study drug (placebo, alteplase 10 mg, or alteplase 20 mg) was manually infused before stent implantation. The drug was reconstituted by the clinical staff using 20 ml of sterile water for injection. The cardiologist then infused the solubilized drug over 5–10 minutes directly into the infarct-related artery proximal to the culprit lesion using either an intra-coronary catheter or the guiding catheter if selectively engaged.

## Secondary Outcomes

## Central laboratory analyses

The central laboratory analyses of the primary and secondary outcomes were determined blind to treatment allocation.

## Cardiac magnetic resonance acquisition and analysis

We used cardiac MRI to assess left ventricular dimensions, function and pathology 2 – 7 days and 3 months post-MI. MRI was performed using 1.5-T platforms (Siemens MAGNETOM Avanto, Erlangen, Germany and Philips Intera, Best, The Netherlands). The imaging protocol followed a Standard Operating Procedure that included planning and localizers, T1-mapping, T2*-mapping, cine MRI with steady-state free precession (SSFP), and late gadolinium enhancement imaging 10 – 15 minutes after administration of contrast media.(2) The scan acquisitions were spatially co-registered and also included different slice orientations to enhance diagnostic confidence.

### Typical T2* imaging parameters

T2* multi-echo GRE (preferred) or T2*-map Bandwidth ~814 (x8) Hz/pixel; flip angle 18°; matrix 256x115 pixels; spatial resolution 2.6 x 1.6 x 10 mm; slice thickness 8 mm with 2 mm gap.

### Contrast media administration

The intravenous contrast agent used in this study was gadobutrol (Gadovist®, Bayer; 1.5 mmol/ml solution for injection) which was administered in two doses in a weight-adjusted contrast volume this was in order to obtain information on myocardial perfusion.

First dose injection (D1 = 0.05 mmol/kg) was given to initiate the first-pass of contrast and the second dose, D2 = 0.1 mmol/kg ‘top-up’ injection, was given immediately after the first-pass. Therefore, the total dose of gadobutrol was 0.15 mmol/kg. An automated pump injector was used for intravenous injection of gadolinium. The injection rate was 4 ml/sec. A 3-of-5 MRI acquisition protocol for short axis imaging of the first pass of gadolinium contrast perfusion was acquired at the same slice positions as the T2* scans. First-pass perfusion at rest for ‘wash-in’ microvascular obstruction quantification was performed with a fast low-angle shot (FLASH) sequence run simultaneously with the contrast injection. ‘Normal’ standard sequence, i.e. non work-in-progress implementation, for 3 short axis (SAX) per R-R interval over 1.5 min was used.

Typical first-pass imaging parameters: Saturation recovery with inversion pulse. T1 101 ms; TR/TE 194/0.98 ms; acquisition window 1000 ms. 1 concatenation; 3 SAX slices. If three slices could not be acquired within the R-R cycle, then 2 concatenations were used.

Cardiac mass & function incorporating area-at-risk imaging with contrast cine-SSFP was acquired 2 – 3 minutes following the second dose of contrast media to enable contrast equilibration. The cine-MRI for LV mass and function was collected during the interval between the 3 min scan and late enhancement imaging. Cine-MRI was acquired with a short axis LV stack, slices aligned to T2* maps, from the mitral valve to the LV apex (usually 10 slices in total). Extra slices were acquired basally to the mitral valve that incorporate left ventricular outflow tract and also potentially to the apex.

Steady-state free precession (SSFP) cine breath-hold sequences (with parallel imaging acceleration) were used. The heart was imaged in multiple parallel SAX planes 8-mm thick separated by 2 mm gaps, equating to approximately 10 slices and 30 cardiac phases.

Typical SSFP imaging parameters: Voxel size 2.0 x 2.0 x 8.0 mm; TR/TE 39.6/1.12 ms; flip angle 55°, matrix 192 x 192 pixels; slice thickness 8 mm, with 2 mm gap.

### Late gadolinium enhancement

Late microvascular obstruction and scar was imaged 10-15 minutes after intravenous Gadovist contrast administration using, in general, a motion-corrected T1-weighted phase-sensitive inversion recovery radiofrequency pulse sequence. A full stack, aligned to T2* scans (or cines), and three long axis views (vertical long axis, horizontal long axis and 3 chamber view) were acquired. Phase-sensitive inversion recovery MRI techniques reduce variability relating to myocardial nulling which is required for late gadolinium enhancement imaging of infarct vs. unaffected myocardium. If a phase-sensitive protocol was not used, a modified Look-Locker inversion time scout was performed prior to using an inversion recovery turbo gradient echo sequence. Phase swaps were performed where appropriate to rule out artefact.

Poor breath-holding: A single shot technique or navigated late gadolinium enhancement imaging was used as an option for poor breath holders.

Typical late gadolinium enhancement and microvascular obstruction imaging parameters with phase-sensitive inversion recovery: matrix 192 x 256 pixels; flip angle 25°; TE 3.36 ms; bandwidth 130 Hz/pixel; echo spacing 8.7ms and trigger pulse 2. The voxel size is 1.8 x 1.3 x 8 mm. Inversion times individually adjusted to optimize nulling of apparently normal myocardium (typical values, 200 to 300 ms).

### Post-contrast T1 mapping

Three short axis T1-maps (basal, mid and apical positions) that were spatially matched to the same slice positions as the pre-contrast T1-map scans >15 minutes after the top-up injection of contrast media.

## Cardiac magnetic resonance imaging at 3 months

Cardiac magnetic resonance imaging (MRI) at 3 months follow-up involved the same scan protocol as was used in the baseline MRI scan. Renal function was known or checked. A full blood count was checked to provide hematocrit for the extracellular volume analysis from the post-contrast T1-map.

## MRI analysis

The MRI analyses were undertaken using Medis® Suite MR (Medis, Leiden, NL) which is a vendor-independent post-processing software. P.McC. undertook the primary analyses of the scans and related analyses were reviewed by C.B. (second observer). The research staff were blinded to treatment allocation.

### Infarct definition and size

The presence of acute infarction was established based on abnormalities in cine wall motion, rest first-pass myocardial perfusion, and late gadolinium enhancement imaging in two imaging planes. The myocardial mass of late gadolinium (grams) was quantified using computer assisted planimetry and the territory of infarction was delineated using a 5 standard deviation method and expressed as a percentage of total left ventricular mass.

### Microvascular obstruction

Microvascular obstruction was defined as a dark zone on early gadolinium enhancement imaging 1, 3, 5, and 7 minutes post-contrast injection that remained present within an area of late gadolinium enhancement at 15 minutes. The myocardial mass (grams) of the dark zone was quantified by manual delineation and expressed as percentage of left ventricular mass.

### Myocardial edema

The presence of myocardial edema was established based on an area of increased signal intensity on the steady-state free precession cine images (acquired two minutes after gadolinium contrast injection). The myocardial mass was calculated by manual delineation in end-diastole and end-systole. The values were averaged and expressed as a percentage of left ventricular mass mass.(2)

### Myocardial salvage

Myocardial salvage was calculated by subtraction of percent infarct size from percent area-at-risk, as reflected by the extent of edema. The myocardial salvage index was calculated by dividing the myocardial salvage area by the initial area-at-risk.

### Myocardial hemorrhage

On the T2* parametric maps, a threshold of 20ms was applied. A region of reduced signal intensity within the infarcted area, with a T2* value of <20 ms (3,4) was considered to confirm the presence of myocardial hemorrhage. The area was manually delineated and expressed as % left ventricular mass.

## Safety

Parameters of hemostasis and coagulation, including fibrinogen concentration, plasminogen activity, Fibrin D-dimer, and Prothrombin F1+2 served as a surrogate measure of bleeding risk and safety.(5,6) TPA antigen levels were assessed as a potential measure of the systemic overflow of the alteplase administered into the culprit coronary artery. Hemostasis and coagulation parameters were measured in blood samples, when site logistics permitted. The sampling time-points were 0 hours, 2, and 24 hours post-reperfusion.

A depletion of fibrinogen and plasminogen following thrombolysis correlates with systemic fibrinolysis and may correlate with bleeding risk. Fibrin D-dimer concentration represents a specific measure of fibrinolysis. Fibrin D-dimer concentrations may correlate with the amount of clot lysis and may therefore represent a measure of residual clot burden. Fibrin D-dimer concentrations have the potential to correlate with efficacy and outcome. Prothrombin F1+2 is a measure of thrombin activation and correlates with the (undesired) procoagulant effect of thrombolysis. F1+2 are depressed by anti-coagulants administered before and during PCI. Prothrombin F1+2 concentrations may associate with the dose of alteplase and placebo and potentially could correlate with adverse thrombotic events.

## Local hospital blood sample handling

Blood samples collected into 0.109M sodium citrate (for hemostasis assays) or EDTA (Troponin) were handled according to a sample handling manual which was provided to all sites. Research blood tests at T 0, 2 hrs. and 24 hrs. were preferred but not compulsory. The blood samples were centrifuged locally and plasma separated and frozen within 2 hours of sampling. Frozen plasma samples were subsequently transported on dry ice for central laboratory analysis in the Department of Hematology, Macewan Building, 16 Alexandra Parade, Glasgow Royal Infirmary, G31 2ER. Plasma samples were stored at -80ºC until analysis, with residual samples being transferred to the Glasgow Biorepository for storage at the end of the study.

## Central laboratory analyses

### Blood samples

Research blood tests at T 0, 2 hrs. and 24 hrs. were preferred but not compulsory. In this way blood sample handling out-of-hours did not become a barrier to enrolment.

### Troponin T

EDTA plasma samples were stored at -80ºC in the Glasgow Royal Infirmary until batch analysis at the end of the study. The biochemical analyses were performed in the British Heart Foundation Glasgow Cardiovascular Research Center.

EDTA plasma samples were stored to analyse high-sensitivity cardiac troponin T on first thaw. Serial measurements of troponin T using the Roche high-sensitivity assay were used to provide a biochemical measurement of infarct size (area-under-the-curve).(7,8) Troponin T (ng/ml) was measured in blood samples collected at baseline (before study drug administration), and 2hr ± 1, 24 hr. ±12, 2-7 days, and 12 weeks (± 2 weeks) post-MI.

For measurement of high sensitivity cardiac troponin T, we used an automated method (e411, Roche Diagnostics, Burgess Hill, United Kingdom) calibrated and quality controlled using the manufacturers reagents. We also participated in the National External Quality Assurance Scheme (NEQAS). The lower limit of detection of Troponin T is 0.003 ng/ml and the 99th percentile value in a healthy subpopulation is 0.0014 ng/ml (Roche Diagnostics, data on file). The between-assay coefficient of variations were 2.2% and 4.2% for control materials with mean Troponin T concentrations of 2.098 ng/ml and 0.0027 ng/ml, respectively.

The troponin T results were provided to the Robertson Center for Biostatistics, University of Glasgow.

## Hemostasis and coagulation laboratory methods

Fibrinogen and other hemostasis parameters served as a surrogate measure of bleeding and safety.(5,6) Hemostasis and coagulation parameters were measured in blood samples when site logistics permitted. The sampling time-points were 0 hours, and 2, and 24 hours post-reperfusion. The parameters included fibrinogen, fibrin D-Dimer and plasminogen activity, tPA antigen and prothrombin F1+2. Fibrinogen circulates in the blood and is converted by thrombin to fibrin leading to clot formation. Plasminogen is the inactive precursor of the serine protease, plasmin. The coagulation parameters that were measured in this study included, fibrinogen and plasminogen (both measures of coagulation and systemic fibrinolysis), fibrin D-dimer (a measure of fibrin lysis), tissue plasminogen activator (a measure of endogenous tPa and any circulating alteplase) and prothrombin F1+2 (a measure of thrombin activation).

### Sample Handling

All plasma samples were processed in a non-standard manner using anonymised bar coded samples by a trained member of staff.

### Assays

Standard laboratory assays (Fibrinogen by Clauss method; high sensitivity Fibrin D-Dimer by latex immunoassay; and Plasminogen Activity by chromogenic assay were performed on an IL TOP700 analyser using HemosIL^®^ reagents (Instrumentation Laboratory Company, Bedford, US). The fibrinogen Clauss assay had a normal reference range 170 – 400 mg/dL (internally derived) and, an inter-assay coefficient of variation of 5.8% and 7.7% for low control samples with mean concentrations of 292 mg/dL and 222 mg/dL respectively. The Fibrin D-Dimer assay had a normal reference range <0.230 μg/ml (manufacturer derived), and an inter-assay coefficient of variation of 11.7 % and 5.2% for control samples with mean concentrations of 0.343 μg/ml and 0.770 μg/ml, respectively. The plasminogen activity assay had a normal reference range 80 – 133 U/dL (manufacturer derived), and an inter-assay coefficient of variation of 2.1% and 1.8% for control samples with mean concentrations of 95.4 U/dL and 29.6 U/dL, respectively.

Non-standard laboratory ELISA assays (tissue plasminogen activator [tPA] and Prothrombin F1+2 antigen levels) were performed on a TECAN Sunrise spectrophotometer (Labtech International Ltd, United Kingdom) using Zymutest tPA Antigen (Hyphen BioMed, Neuville-sur-oise, France)) and Enzygnost F1+2 Mono (Siemens, Marburg, Germany) commercial kits respectively. The tPA antigen assay had a normal reference range <10 ng/ml (manufacturer derived), and an inter-assay coefficient of variation of 4.7% and 11% for control samples with mean concentrations of 11.0 ng/ml and 3.1 ng/ml, respectively. The F1+2 assay had a normal reference range 69 – 229 pmol/L (manufacturer derived) and, an inter-assay coefficient of variation of 7.9% for a normal control sample with a mean concentration of 97.6 pmol/L.

## Trial management

The trial was conducted in line with Guidelines for Good Clinical Practice in Clinical Trials(9) and the study complies with the Declaration of Helsinki.(10) There was a Trial Management Group for operational activity, an independent Clinical Event Committee to adjudicate on serious adverse events for safety and efficacy outcomes, an independent Data and Safety Monitoring Committee and a Trial Steering Committee to coordinate the trial and liaise with the Sponsor and Trials Unit. Each committee had a charter that was established before enrolment started

The independent Data and Safety Monitoring Committee met before the enrolment began, and twice again during the active phase of the trial. This committee had responsibility for potentially recommending early discontinuation of the entire study or an individual arm because of safety concerns or due to futility. The funder, the Efficacy and Mechanism Evaluation (EME) program of the National Institute for Health Research (NIHR) required an interim analysis for futility and also specified the criteria. This analysis was scheduled for when approximately 40% of patients had been randomized and followed-up to 3 months. Considering the primary outcome, each active treatment arm was compared to the placebo arm and if the conditional power for showing a benefit over placebo based on the current trend was less than 30%, then a recommendation would be made to halt that arm.

The Robertson Center for Biostatistics within the Glasgow Clinical Trials Unit provided the trial-specific electronic data collection system, acted as an independent coordinating center for randomization and data management, and conducted the statistical analyses. The trial was approved by the National Research Ethics Service (reference 13/WS/0119). The clinical trial registration number is NCT02257294 and the trial was co-sponsored by the University of Glasgow and Greater Glasgow and Clyde Health Board, NHS Scotland. The sponsor undertook feasibility assessments at each site. The sponsor prospectively monitored the study for safety and monitoring, visits were undertaken in all of the sites. All serious adverse events were prospectively reported to the Pharmacovigilance Unit.

## Statistical methods

Secondary outcomes were summarized in the final analysis set as a whole and by treatment group. All outcomes were compared between treatment groups using linear, binary logistic or proportional odds logistic regression models, for continuous, binary, and ordinal outcomes respectively, with adjustment for location of the MI. To maximize power, randomized treatment was modelled as a linear trend across dose groups (0mg, 10mg, 20mg), though analyses treating randomized treatment as a three-level categorical variable, or as a two-level categorical variable (active vs. placebo) were also performed. Treatment was initially included as a 3-level categorical variable, and each treatment group was compared with placebo; in a second model, treatment was included as a binary variable, to compare both alteplase groups combined with placebo. For continuous outcome measures, model residual distributions were examined and outcomes were transformed for modelling where necessary to improve model fit.

Treatment effect estimates were reported with 95% confidence intervals (CIs) and p-values. Where no suitable transformation was found, each active treatment group was compared to placebo using van Elteren tests, stratified by the location of the MI. Ordinal outcomes were compared between groups using proportional odds logistic regression models, adjusted for the location of the MI. Binary outcomes were compared between groups using logistic regression models, adjusted for the location of the MI. Logistic regression model results are reported as odds ratios for each active treatment group vs. placebo, with 95% confidence intervals and p-values. For those outcomes measured at both 2 – 7 days and at 3 months, changes between the two time points were summarized, and regression models of 3 month outcomes were extended to include an adjustment for the day 2 – 7 measurement. All statistical analyses were carried out with or R v3.2.4 [R Development Core Team 2015].(11) The statistical analyses were conducted according to a pre-specified Statistical Analysis Plan (SAP), which was authored by the Trial Statistician and agreed by the Trial Steering Committee. The SAP was approved, and all statistical analysis programs were written and validated prior to database lock, at which point the randomized treatment allocations were released.

# References

1. Ibanez B, James S, Agewall S et al. 2017 ESC Guidelines for the management of acute myocardial infarction in patients presenting with ST-segment elevation: The Task Force for the management of acute myocardial infarction in patients presenting with ST-segment elevation of the European Society of Cardiology (ESC). Eur Heart J 2018;39:119-177.

2. Sorensson P, Heiberg E, Saleh N et al. Assessment of myocardium at risk with contrast enhanced steady-state free precession cine cardiovascular magnetic resonance compared to single-photon emission computed tomography. J Cardiovasc Magn Reson 2010;12:25.

3. Anderson LJ, Holden S, Davis B et al. Cardiovascular T2-star (T2*) magnetic resonance for the early diagnosis of myocardial iron overload. Eur Heart J 2001;22:2171-9.

4. Carrick D, Haig C, Ahmed N et al. Myocardial Hemorrhage After Acute Reperfused ST-Segment-Elevation Myocardial Infarction: Relation to Microvascular Obstruction and Prognostic Significance. Circ Cardiovasc Imaging 2016;9:e004148.

5. Rao AK, Pratt C, Berke A et al. Thrombolysis in Myocardial Infarction (TIMI) Trial--phase I: hemorrhagic manifestations and changes in plasma fibrinogen and the fibrinolytic system in patients treated with recombinant tissue plasminogen activator and streptokinase. J Am Coll Cardiol 1988;11:1-11.

6. Huang X, Moreton FC, Kalladka D et al. Coagulation and Fibrinolytic Activity of Tenecteplase and Alteplase in Acute Ischemic Stroke. Stroke 2015;46:3543-6.

7. Thygesen K, Mair J, Giannitsis E et al. How to use high-sensitivity cardiac troponins in acute cardiac care. Eur Heart J 2012;33:2252-7.

8. Steg PG, James SK, Atar D et al. ESC Guidelines for the management of acute myocardial infarction in patients presenting with ST-segment elevation. Eur Heart J 2012;33:2569-619.

9. <https://www.gov.uk/guidance/good-clinical-practice-for-clinical-trials>. Accessed January 13, 2019.

10. World Medical Association Declaration of Helsinki: ethical principles for medical research involving human subjects. Jama 2013;310:2191-4.

11. R Core Team (2016). R: A language and environment for statistical computing. https://www.R-project.org. Accessed January 13, 2019.

# Supplementary Tables.

## Supplementary Table I. Baseline clinical characteristics, ischemic time and treatment of the randomized participants (n=440).

|  | **Randomly assigned** | | |
| --- | --- | --- | --- |
| Baseline characteristics | Placebo  (n = 151) | Alteplase, 10 mg  (n = 144) | Alteplase, 20 mg  (n = 145) |
| Clinical |  |  |  |
| Age, years | 60.7 (11.0) | 59.6 (10.3) | 61.2 (9.7) |
| Male sex, n (%) | 127 (84.1) | 124 (86.1) | 123 (84.8) |
| Race, white, n (%) | 143 (94.7) | 134 (93.1) | 136 (93.8) |
| Body mass index, kg/m^2^ | 28.4 (5.3) | 28.5 (4.8) | 27.8 (4.4) |
| Presenting characteristics |  |  |  |
| Heart rate, bpm | 73.3 (22.5) | 71.8 (15.9) | 73.5 (17.6) |
| Systolic blood pressure, mmHg | 132 (26) | 135 (25) | 134 (25) |
| Diastolic blood pressure, mmHg | 79 (17) | 80 (15) | 81 (15) |
| Infarct location, n (%) |  |  |  |
| Anterior | 65 (43.0) | 62 (43.1) | 64 (44.1) |
| Inferior | 70 (46.4) | 67 (46.5) | 70 (48.3) |
| Lateral | 1 (0.7) | 2 (1.4) | 0 (0) |
| Posterior | 14 (9.3) | 11 (7.6) | 8 (5.5) |
| Other | 1 (0.7) | 2 (1.4) | 3 (2.1) |
| Medical history |  |  |  |
| Hypertension*, n (%) | 47 (31.1) | 45 (31.2) | 49 (33.8) |
| Diabetes mellitus*, n (%) | 19 (12.6) | 19 (13.2) | 18 (12.4) |
| Hypercholesterolemia*, n (%) | 42 (27.8) | 28 (19.4) | 32 (22.1) |
| Smoking*, n (%) |  |  |  |
| Current | 75 (49.7) | 72 (50.0) | 62 (42.8) |
| Former (stopped > 3 months) | 27 (17.9) | 22 (15.3) | 35 (24.1) |
| Never | 49 (32.5) | 50 (34.7) | 48 (33.1) |
| Percutaneous coronary intervention, n (%) | 8 (5.3) | 5 (3.5) | 7 (4.8) |
| Coronary artery bypass graft surgery, n (%) | 0 (0) | 0 (0) | 0 (0) |
| Angina, n (%) | 6 (4.0) | 7 (4.9) | 4 (2.8) |
| Myocardial infarction, n (%) | 6 (4.0) | 6 (4.2) | 8 (5.5) |
| Stroke or transient ischemic attack*, n (%) | 2 (1.3) | 1 (0.7) | 2 (1.4) |
| Peripheral vascular disease*, n (%) | 3 (2.0) | 3 (2.1) | 6 (4.1) |
| Pre-existing maintenance medication |  |  |  |
| Aspirin, n (%) | 27 (17.9) | 17 (11.8) | 22 (15.2) |
| P2Y12 inhibitor, n (%) |  |  |  |
| Clopidogrel | 1 (0.7) | 0 (0.0) | 1 (0.7) |
| Ticagrelor or Prasugrel | 9 (6.0) | 4 (2.8) | 7 (4.8) |
| Statin, n (%) | 40 (26.5) | 29 (20.1) | 28 (19.3) |
| Beta-blocker, n (%) | 17 (11.3) | 15 (10.4) | 10 (6.9) |
| ACE-I or ARB, n (%) | 23 (15.2) | 28 (19.5) | 27 (18.6) |
| Mineralocorticoid receptor antagonist, n (%) | 1 (0.7) | 2 (1.4) | 1 (0.7) |
| Symptom onset to arrival at primary PCI center, hours:mins | 2:05 (1:34, 3:01) | 2:11 (1:31, 3:26) | 2:15 (1:32, 3:15) |
| Arrival at primary PCI center to reperfusion, mins | 24 (19, 35) | 23 (19, 37) | 25 (19, 34) |
| Symptom onset to reperfusion, hours:mins | 2:36 (2:03, 3:36) | 2:50 1:55, 4:06) | 2:44 (2:01, 3:49) |
| Ischemic time |  |  |  |
| <2 hours, n (%) | 32 (21.2) | 42 (29.2) | 33 (22.8) |
| ≥2, <4 hours, n (%) | 90 (59.6) | 64 (44.4) | 81 (55.9) |
| ≥4 - 6 hours, n (%) | 29 (19.2) | 38 (26.4) | 31 (21.4) |
| Initial blood results on admission |  |  |  |
| Haemoglobin, g/L | 144.0 (14.1) | 145.9 (12.4) | 146.6 (13.2) |
| Platelet count, x10^9^L | 253.7 (59.8) | 267.9 (72.0) | 260.4 (53.3) |
| Creatinine, μmol/L | 80 (18) | 80 (17) | 80 (18) |
| Troponin, ng/L | 60 (27, 128) | 57 (32, 101) | 59 (31, 120) |

Footnote: data summarised as mean±SD or median (interquartile range) for normal and non-normally distributed data respectively. * At least one risk factor for coronary artery disease was required for eligibility. Diabetes mellitus was defined as a history of diet-controlled or treated diabetes. ACE-I = angiotensin converting enzyme inhibitor; ARB = angiotensin receptor blocker; IQR = interquartile range; PCI = percutaneous coronary intervention

## Table II. Pre-specified analyses of the secondary outcomes at 3 months, adjusting for location of MI, by sub-groups of ischemic time and interactions with treatment, effect estimates and interactions.

| **Table II. Secondary outcomes by randomized treatment, by sub-groups of ischemic time, with tests for differences between treatment groups** | | | | | | | | |
| --- | --- | --- | --- | --- | --- | --- | --- | --- |
|  |  |  | Treatment Effect  (Alteplase 20mg vs. Alteplase 10mg vs. Placebo) | | |  | Treatment Effect  (Alteplase vs. Placebo) | |
| Outcome | | | 10mg vs. Placebo  Estimate (95% CI), p-value | 20mg vs. Placebo  Estimate (95% CI), p-value | Interaction p-value |  | Estimate (95% CI), p-value | Interaction p-value |
|  | LV ejection fraction at 3 months. Treatment effects reported as mean differences between groups | | | | | | | |
|  |  | Ischemic Time <2 hours  Ischemic Time ≥2, <4 hours  Ischemic Time ≥4 - 6 hours | -1.7 (-5.9, 2.4), p=0.408  -0.2 (-3.1, 2.7), p=0.887  -4.8 (-9.2, -0.3), p=0.037 | -1.9 (-6.3, 2.5), p=0.401  -0.2 (-2.9, 2.5), p=0.900  -3.8 (-8.4, 0.7), p=0.100 | p=0.514 |  | -1.8 (-5.5, 1.9), p=0.347  -0.2 (-2.6, 2.2), p=0.873  -4.3 (-8.2, -0.4), p=0.030 | p=0.204 |
|  | LV end-systolic volume at 3 months. Data analyzed on a logarithmic scale; treatment effects reported as relative differences between groups | | | | | | | |
|  |  | Ischemic Time <2 hours  Ischemic Time ≥2, <4 hours  Ischemic Time ≥4 - 6 hours | 1.22 (1.02, 1.46), p=0.029  1.00 (0.88, 1.13), p=0.970  1.18 (0.97, 1.44), p=0.089 | 1.11 (0.92, 1.35), p=0.272  0.96 (0.85, 1.08), p=0.467  1.16 (0.95, 1.42), p=0.134 | p=0.246 |  | 1.17 (1.00, 1.38), p=0.054  0.97 (0.88, 1.08), p=0.626  1.17 (0.99, 1.39), p=0.062 | p=0.066 |
|  | LV end-diastolic volume at 3 months. Data analyzed on a logarithmic scale; treatment effects reported as relative differences between groups | | | | | | | |
|  |  | Ischemic Time <2 hours  Ischemic Time ≥2, <4 hours  Ischemic Time ≥4 - 6 hours | 1.17 (1.04, 1.32), p=0.009  1.01 (0.93, 1.10), p=0.863  1.08 (0.95, 1.23), p=0.238 | 1.07 (0.94, 1.21), p=0.302  0.94 (0.87, 1.02), p=0.151  1.08 (0.95, 1.23), p=0.242 | p=0.158 |  | 1.13 (1.01, 1.26), p=0.031  0.97 (0.91, 1.04), p=0.407  1.08 (0.97, 1.21), p=0.173 | p=0.047 |
| Myocardial salvage (% LV) at 3 months. Data analyzed on original scale; treatment effect reported as relative increase per 10mg increase in alteplase dose | | | | | | | | |
|  |  | Ischemic Time <2 hours  Ischemic Time ≥2, <4 hours  Ischemic Time ≥4 - 6 hours | -0.02 (-0.13, 0.10), p=0.750  0.05 (-0.03, 0.13), p=0.205  -0.09 (-0.22, 0.03), p=0.150 | -0.03 (-0.16, 0.09), p=0.609  0.01 (-0.07, 0.08), p=0.860  -0.10 (-0.22, 0.03), p=0.133 | p=0.398 |  | -0.02 (-0.13, 0.08), p=0.650  0.03 (-0.04, 0.09), p=0.443  -0.09 (-0.20, 0.01), p=0.088 | p=0.172 |
| Infarct size (% LV) at 3 months. Data analyzed on a logarithmic scale; | | | | | | | | |
|  |  | Ischemic Time <2 hours  Ischemic Time ≥2, <4 hours  Ischemic Time ≥4 - 6 hours | 1.69 (-3.92, 7.30), p=0.554  -1.51 (-5.42, 2.41), p=0.451  2.96 (-3.06, 8.97), p=0.335 | 2.98 (-2.99, 8.95), p=0.3277  -0.27 (-3.92, 3.38), p=0.883  2.07 (-4.05, 8.19), p=0.507 | p=0.720 |  | 2.24 (-2.88, 7.36), p=0.391  -0.80 (-4.03, 2.43), p=0.626  2.53 (-2.69, 7.75), p=0.342 | p=0.440 |

Footnote: All outcomes were pre-specified. Treatment effect estimates derived from linear or logistic regression models, modelling the treatment effect as a linear trend across alteplase dose groups (0mg vs. 10mg vs. 20mg). Interaction test p-values reported from regression models with ischemic time included as a 3-level categorical variable, and interaction with treatment effect.

## Supplementary Table III. Pre-specified analyses of secondary outcomes for hematology and coagulation, adjusting for location of MI, by sub-groups of ischemic time and interactions between treatment (active vs. placebo).

| **Table III. Secondary outcomes by randomized treatment, by sub-groups of ischemic time, with tests of trend in relation to alteplase dose** | | | | | | | | | |
| --- | --- | --- | --- | --- | --- | --- | --- | --- | --- |
|  |  |  |  | Randomized Treatment Group | | |  | Treatment Effect  (Trend with dose) | |
| Outcome | | | N (missing) | Placebo  (n=151) | Alteplase, 10 mg  (n=144) | Alteplase, 20 mg  (n=145) |  | Estimate (95% CI), p-value | Interaction p-value |
|  | Fibrin D-Dimers (ng/ml) 2 hours. Data analyzed on a logarithmic scale; treatment effect reported as ratios per 10mg increase in alteplase dose | | | | | | | | |
|  |  | Ischemic Time <2 hours  Ischemic Time ≥2, <4 hours  Ischemic Time ≥4 - 6 hours | 93 (14)  185 (50)  80 (18) | 100 (73, 160)  118 (75, 157)  114 (65, 145) | 261 (151, 496)  406 (273, 671)  348 (261, 613) | 421 (282, 1008)  418 (262, 640)  445 (360, 757) |  | 2.11 (1.69, 2.65), p<0.001  1.97 (1.70, 2.27), p<0.001  2.32 (1.83, 2.95), p<0.001 | p=0.494 |
|  | Ratio of fibrin D-Dimers (2 hours) to baseline. Data analyzed on a logarithmic scale; treatment effect reported as ratios per 10mg increase in alteplase dose | | | | | | | | |
|  |  | Ischemic Time <2 hours  Ischemic Time ≥2, <4 hours  Ischemic Time ≥4 - 6 hours | 91 (13)  181 (53)  79 (18) | 1.06 (0.94, 1.33)  1.10 (0.96, 1.32)  1.08 (0.96, 1.34) | 2.37 (1.68, 3.39)  3.53 (2.88, 4.95)  3.99 (2.56, 6.17) | 4.96 (2.57, 8.33)  3.91 (2.55, 6.66)  4.51 (3.70, 6.94) |  | 2.05 (1.72, 2.44), p<0.001  1.96 (1.75, 2.19), p<0.001  2.01 (1.67, 2.41), p<0.001 | p=0.909 |
|  | Prothrombin F1+2 (pmol/l) 2 hours. Data analyzed on a logarithmic scale; treatment effect reported as ratios per 10mg increase in alteplase dose | | | | | | | | |
|  |  | Ischemic Time <2 hours  Ischemic Time ≥2, <4 hours  Ischemic Time ≥4 - 6 hours | 92 (12)  185 (49)  80 (17) | 147 (125, 206)  185 (136, 267)  150 (122, 186) | 158 (134, 261)  200 (156, 239)  194 (139, 330) | 193 (136, 274)  192 (152, 296)  226 (174, 326) |  | 1.11 (0.94, 1.30), p=0.209  1.06 (0.96, 1.17), p=0.277  1.21 (1.03, 1.44), p=0.024 | p=0.385 |
|  | Ratio of Prothrombin F1+2 (pmol/l) 2 hours to baseline. Data analyzed on a logarithmic scale; treatment effect reported as ratios per 10mg increase in alteplase dose | | | | | | | | |
|  |  | Ischemic Time <2 hours  Ischemic Time ≥2, <4 hours  Ischemic Time ≥4 - 6 hours | 91 (13)  181 (53)  79 (18) | 0.94 (0.79, 1.07)  1.09 (0.96, 1.37)  1.06 (0.92, 1.37) | 1.19 (1.02, 1.42)  1.24 (0.98, 1.58)  1.21 (1.05, 1.93) | 1.23 (0.96, 1.54)  1.27 (1.06, 1.59)  1.35 (1.11, 1.71) |  | 1.17 (1.01, 1.34), p=0.032  1.08 (0.99, 1.19), p=0.080  1.13 (0.98, 1.31), p=0.097 | p=0.660 |
|  | tPA (ng/ml) on day of procedure (2 hours). Data analyzed on a logarithmic scale; treatment effect reported as ratios per 10mg increase in alteplase dose | | | | | | | | |
|  |  | Ischemic Time <2 hours  Ischemic Time ≥2, <4 hours  Ischemic Time ≥4 - 6 hours | 92 (12)  185 (49)  80 (17) | 10.0 (8.0, 13.0)  11.0 (9.0, 13.0)  10.0 (9.0, 13.8) | 12.5 (11.0, 15.2)  13.0 (11.0, 16.0)  13.5 (12.0, 18.0) | 15.0 (14.0, 19.5)  14.0 (12.0, 16.0)  12.0 (12.0, 18.5) |  | 1.32 (1.15, 1.51), p<0.001  1.21 (1.10, 1.31), p<0.001  1.16 (1.00, 1.33), p=0.050 | p=0.105 |
|  | Ratio of tPA (ng/ml) 2 hours to baseline. Data analyzed on a logarithmic scale; treatment effect reported as ratios per 10mg increase in alteplase dose | | | | | | | | |
|  |  | Ischemic Time <2 hours  Ischemic Time ≥2, <4 hours  Ischemic Time ≥4 - 6 hours | 91 (13)  181 (53)  79 (18) | 1.04 (0.88, 1.25)  1.10 (1.00, 1.26)  1.13 (1.00, 1.28) | 1.37 (1.17, 1.61)  1.31 (1.17, 1.67)  1.30 (1.20, 1.69) | 1.62 (1.35, 2.00)  1.44 (1.21, 1.79)  1.57 (1.43, 2.06) |  | 1.16 (0.97, 1.38), p=0.199  1.11 (1.00, 1.25), p=0.059  1.30 (1.08, 1.57), p=0.005 | p=0.360 |
|  | Plasminogen (U/dL) on day of procedure (2 hours). Treatment effects reported as mean differences per 10mg increase in alteplase dose | | | | | | | | |
|  |  | Ischemic Time <2 hours  Ischemic Time ≥2, <4 hours  Ischemic Time ≥4 - 6 hours | 92 (12)  185 (49)  80 (17) | 93.3 (15.6)  94.9 (12.8)  95.3 (15.0) | 90.1 (11.8)  88.8 (12.8)  89.6 (12.5) | 81.9 (16.2)  84.3 (11.9)  86.1 (14.1) |  | -5.74 (-9.23, -2.26), p=0.001  -5.17 (-7.38, -2.95), p<0.001  -4.61 (-8.29, -0.92), p=0.014 | p=0.908 |
|  | Fibrinogen (g/L) on day of procedure (2 hours). Data analyzed on a logarithmic scale; treatment effect reported as ratios per 10mg increase in alteplase dose | | | | | | | | |
|  |  | Ischemic Time <2 hours  Ischemic Time ≥2, <4 hours  Ischemic Time ≥4 - 6 hours | 92 (12)  185 (49)  80 (17) | 3.34 (0.84)  3.46 (0.82)  3.45 (1.16) | 3.17 (0.80)  3.24 (1.21)  3.70 (1.25) | 3.12 (0.58)  3.23 (0.66)  3.39 (0.63) |  | 0.97 (0.91, 1.04), p=0.395  0.97 (0.93, 1.01), p=0.206  1.01 (0.94, 1.09), p=0.718 | p=0.599 |

Footnote: All outcomes were pre-specified. Data summarized as mean±SD or median (interquartile range) for normal and non-normally distributed data respectively. Between-group comparison p-values derived from linear, binary logistic, or proportional odds logistic regression model. Fibrinogen, plasminogen, fibrin D-dimers, prothrombin F1+2, and tissue plasminogen activator were available in 357 patients at 2 hours. tPA = Tissue plasminogen activator.

## Supplementary Table IV. Pre-specified analyses of secondary outcomes for hematology and coagulation, adjusting for location of MI, by sub-groups of ischemic time and interactions between treatment (active vs. placebo), effect estimates and trends of interaction.

| **Table IV. Primary and secondary outcomes by randomized treatment, by sub-groups of ischemic time, with tests for differences between treatment groups** | | | | | | | | | | | | | | |
| --- | --- | --- | --- | --- | --- | --- | --- | --- | --- | --- | --- | --- | --- | --- |
|  |  |  | | Treatment Effect  (Alteplase 20mg vs. Alteplase 10mg vs. Placebo) | | | | | |  | | Treatment Effect  (Alteplase vs. Placebo) | | |
| Outcome | | | 10mg vs. Placebo  Estimate (95% CI), p-value | | 20mg vs. Placebo  Estimate (95% CI), p-value | | Interaction p-value | |  | | Estimate (95% CI), p-value | | Interaction p-value | |
|  | Fibrin D-Dimers (ng/ml) 2 hours. Data analyzed on a logarithmic scale; treatment effects reported as ratios between groups | | | | | | | | | | | | | |
|  |  | Ischemic Time <2 hours  Ischemic Time ≥2, <4 hours  Ischemic Time ≥4 - 6 hours | | 2.20 (1.48, 3.29), p<0.001  3.96 (2.92, 5.37), p<0.001  4.67 (3.03, 7.20), p<0.001 | | 4.47 (2.90, 6.89), p<0.001  3.84 (2.92, 5.06), p<0.001  5.27 (3.34, 8.32), p<0.001 | | p=0.020 | |  | | 2.97 (2.06, 4.28), p<0.001  3.89 (3.04, 4.99), p<0.001  4.92 (3.33, 7.27), p<0.001 | | p=0.177 |
|  | Ratio of fibrin D-Dimers on day of procedure (2 hours) to baseline. Data analyzed on a logarithmic scale; treatment effects reported as ratios between groups | | | | | | | | | | | | | |
|  |  | Ischemic Time <2 hours  Ischemic Time ≥2, <4 hours  Ischemic Time ≥4 - 6 hours | | 2.30 (1.70, 3.13), p<0.001  3.29 (2.61, 4.15), p<0.001  3.98 (2.86, 5.53), p<0.001 | | 4.18 (3.01, 5.82), p<0.001  3.80 (3.08, 4.69), p<0.001  3.93 (2.78, 5.56), p<0.001 | | p=0.046 | |  | | 2.96 (2.24, 3.93), p<0.001  3.58 (2.96, 4.34), p<0.001  3.96 (2.94, 5.33), p<0.001 | | p=0.358 |
|  | Prothrombin F1+2 (pmol/l) 2 hours. Treatment effects reported as ratios between groups | | | | | | | | | | | | | |
|  |  | Ischemic Time <2 hours  Ischemic Time ≥2, <4 hours  Ischemic Time ≥4 - 6 hours | | 1.24 (0.93, 1.67), p=0.146  1.29 (1.03, 1.61), p=0.025  1.32 (0.96, 1.81), p=0.089 | | 1.22 (0.89, 1.67), p=0.216  1.12 (0.91, 1.37), p=0.282  1.47 (1.05, 2.05), p=0.024 | | p=0.663 | |  | | 1.23 (0.95, 1.61), p=0.120  1.18 (0.99, 1.42), p=0.064  1.38 (1.04, 1.83), p=0.024 | | p=0.661 |
|  | Ratio of Prothrombin F1+2 (pmol/l) on day of procedure (2 hours) to baseline. Data analyzed on a logarithmic scale; treatment effects reported as ratios between groups | | | | | | | | | | | | | |
|  |  | Ischemic Time <2 hours  Ischemic Time ≥2, <4 hours  Ischemic Time ≥4 - 6 hours | | 1.38 (1.06, 1.79), p=0.015  1.28 (1.05, 1.56), p=0.014  1.31 (0.99, 1.74), p=0.056 | | 1.36 (1.03, 1.80), p=0.032  1.17 (0.98, 1.40), p=0.082  1.17 (0.98, 1.40), p=0.100 | | p=0.933 | |  | | 1.37 (1.08, 1.73), p=0.008  1.21 (1.04, 1.42), p=0.016  1.30 (1.01, 1.66), p=0.034 | | p=0.692 |
|  | tPA (ng/ml) on day of procedure (2 hours). Data analyzed on a logarithmic scale; treatment effects reported as ratios between groups | | | | | | | | | | | | | |
|  |  | Ischemic Time <2 hours  Ischemic Time ≥2, <4 hours  Ischemic Time ≥4 - 6 hours | | 1.29 (1.00, 1.66), p=0.051  1.26 (1.04, 1.52), p=0.020  1.40 (1.07, 1.84), p=0.016 | | 1.74 (1.32, 2.29), p<0.001  1.45 (1.22, 1.73), p<0.001  1.33 (0.99, 1.77), p=0.056 | | p=0.438 | |  | | 1.46 (1.16, 1.84), p=0.001  1.37 (1.17, 1.60), p<0.001  1.37 (1.07, 1.75), p=0.013 | | p=0.889 |
|  | Ratio of tPA (ng/ml) on day of procedure (2 hours) to baseline Data analyzed on a logarithmic scale; treatment effects reported as ratios between groups | | | | | | | | | | | | | |
|  |  | Ischemic Time <2 hours  Ischemic Time ≥2, <4 hours  Ischemic Time ≥4 - 6 hours | | 1.13 (0.81, 1.57), p=0.465  1.18 (0.92, 1.52), p=0.181  1.26 (0.89, 1.80), p=0.195 | | 1.34 (0.94, 1.91), p=0.103  1.24 (0.99, 1.55), p=0.061  1.70 (1.17, 2.46), p=0.005 | | p=0.669 | |  | | 1.21 (0.90, 1.63), p=0.199  1.22 (1.00, 1.49), p=0.055  1.45 (1.06, 1.98), p=0.021 | | p=0.634 |
|  | Plasminogen (U/dL) on day of procedure (2 hours). Treatment effects reported as mean differences between groups | | | | | | | | | | | | | |
|  |  | Ischemic Time <2 hours  Ischemic Time ≥2, <4 hours  Ischemic Time ≥4 - 6 hours | | -3.32 (-9.79, 3.16), p=0.316  -5.90 (-10.83, -0.98) p=0.019  -5.83 (-12.83, 1.17), p=0.103 | | -11.57(-18.55, -4.58)p=0.001  -10.33(-14.78, -5.89)p=0.363  -9.17 (-16.56, -1.78) p=0.015 | | p=0.875 | |  | | -6.80 (-12.70, -0.90) p=0.024  -8.52 (-12.52, -4.53) p<0.001  -7.31 (-13.60, -1.02) p=0.023 | | p=0.878 |
| Fibrinogen (g/L) on day of procedure (2 hours). Data analyzed on a logarithmic scale; treatment effect reported as ratios between groups | | | | | | | | | | | | | | |
|  |  | Ischemic Time <2 hours  Ischemic Time ≥2, <4 hours  Ischemic Time ≥4 - 6 hours | | 0.94 (0.83, 1.06), p=0.301  0.92 (0.84, 1.01), p=0.088  1.08 (0.95, 1.23), p=0.252 | | 0.95 (0.83, 1.08), p=0.405  0.95 (0.87, 1.03), p=0.209  1.02 (0.89, 1.18), p=0.742 | | p=0.424 | |  | | 0.94 (0.85, 1.05), p=0.301  0.94 (0.87, 1.01), p=0.088  1.05 (0.94, 1.19), p=0.373 | | p=0.228 |

Footnote: All outcomes were pre-specified. Treatment effect estimates derived from linear or logistic regression models, modelling the treatment effect as a linear trend across alteplase dose groups (0mg vs. 10mg vs. 20mg). Interaction test p-values reported from regression models with ischemic time included as a 3-level categorical variable, and interaction with treatment effect. Fibrinogen, plasminogen, fibrin D-dimers, prothrombin F1+2, and tissue plasminogen activator were available in 357 patients at 2 hours. tPA = Tissue plasminogen activator.
